# Selectivity filter mutations shift ion permeation mechanism in potassium channels

**DOI:** 10.1093/pnasnexus/pgae272

**Published:** 2024-07-05

**Authors:** Andrei Mironenko, Bert L de Groot, Wojciech Kopec

**Affiliations:** Computational Biomolecular Dynamics Group, Max Planck Institute for Multidisciplinary Sciences, Am Fassberg 11, Göttingen 37077, Germany; Computational Biomolecular Dynamics Group, Max Planck Institute for Multidisciplinary Sciences, Am Fassberg 11, Göttingen 37077, Germany; Computational Biomolecular Dynamics Group, Max Planck Institute for Multidisciplinary Sciences, Am Fassberg 11, Göttingen 37077, Germany; Department of Chemistry, Queen Mary University of London, 327 Mile End Road, London E1 4NS, UK

**Keywords:** potassium channels, molecular dynamics, ion permeation, permeation mechanisms

## Abstract

Potassium (K^+^) channels combine high conductance with high ion selectivity. To explain this efficiency, two molecular mechanisms have been proposed. The “direct knock-on” mechanism is defined by water-free K^+^ permeation and formation of direct ion–ion contacts in the highly conserved selectivity filter (SF). The “soft knock-on” mechanism involves co-permeation of water and separation of K^+^ by water molecules. With the aim to distinguish between these mechanisms, crystal structures of the KcsA channel with mutations in two SF residues—G77 and T75—were published, where the arrangements of K^+^ ions and water display canonical soft knock-on configurations. These data were interpreted as evidence of the soft knock-on mechanism in wild-type channels. Here, we test this interpretation using molecular dynamics simulations of KcsA and its mutants. We show that while a strictly water-free direct knock-on permeation is observed in the wild type, conformational changes induced by these mutations lead to distinct ion permeation mechanisms, characterized by co-permeation of K^+^ and water. These mechanisms are characterized by reduced conductance and impaired potassium selectivity, supporting the importance of full dehydration of potassium ions for the hallmark high conductance and selectivity of K^+^ channels. In general, we present a case where mutations introduced at the critical points of the permeation pathway in an ion channel drastically change its permeation mechanism in a nonintuitive manner.

**Significance Statement**

Potassium (K^+^) channels conduct K^+^ with high permeation rates and ion selectivity. An ongoing debate in the field has been focused on the molecular mechanisms underlying this remarkable efficiency. Here, we performed molecular dynamics simulations of two selectivity filter (SF) mutants of a model K^+^ channel to investigate this question. These mutations led to a substantial decrease in conductance and ion selectivity, but accompanied by a shift from water-free K^+^ permeation to co-permeation of water and K^+^. Our findings not only provide a fundamental example of how single point mutations in the SF can alter the ion permeation mechanism, but also reinforce the notion that water exclusion underlies the remarkable efficiency of K^+^ channels.

## Introduction

Potassium channels (K^+^ channels) enable the permeation of K^+^ ions along their electrochemical gradient across plasma and organelle membranes in almost all organisms. They play an essential role in establishing the membrane potential in living cells, in terminating action potentials in excitable cells, as well as in many other physiological processes (1). K^+^ channels are able to conduct K^+^ with very high permeation rates, with conductances reaching hundreds of pS (2). At the same time, they possess exceptional selectivity for K^+^ over other monovalent ions, with K^+^/Na^+^ permeability ratios reaching ∼100–1,000 (3). In the last decades, a great effort has been put into determining the ion permeation mechanism that can explain such high K^+^ permeation efficiency, combined with strict K^+^ selectivity (4).

**Fig. 1.**
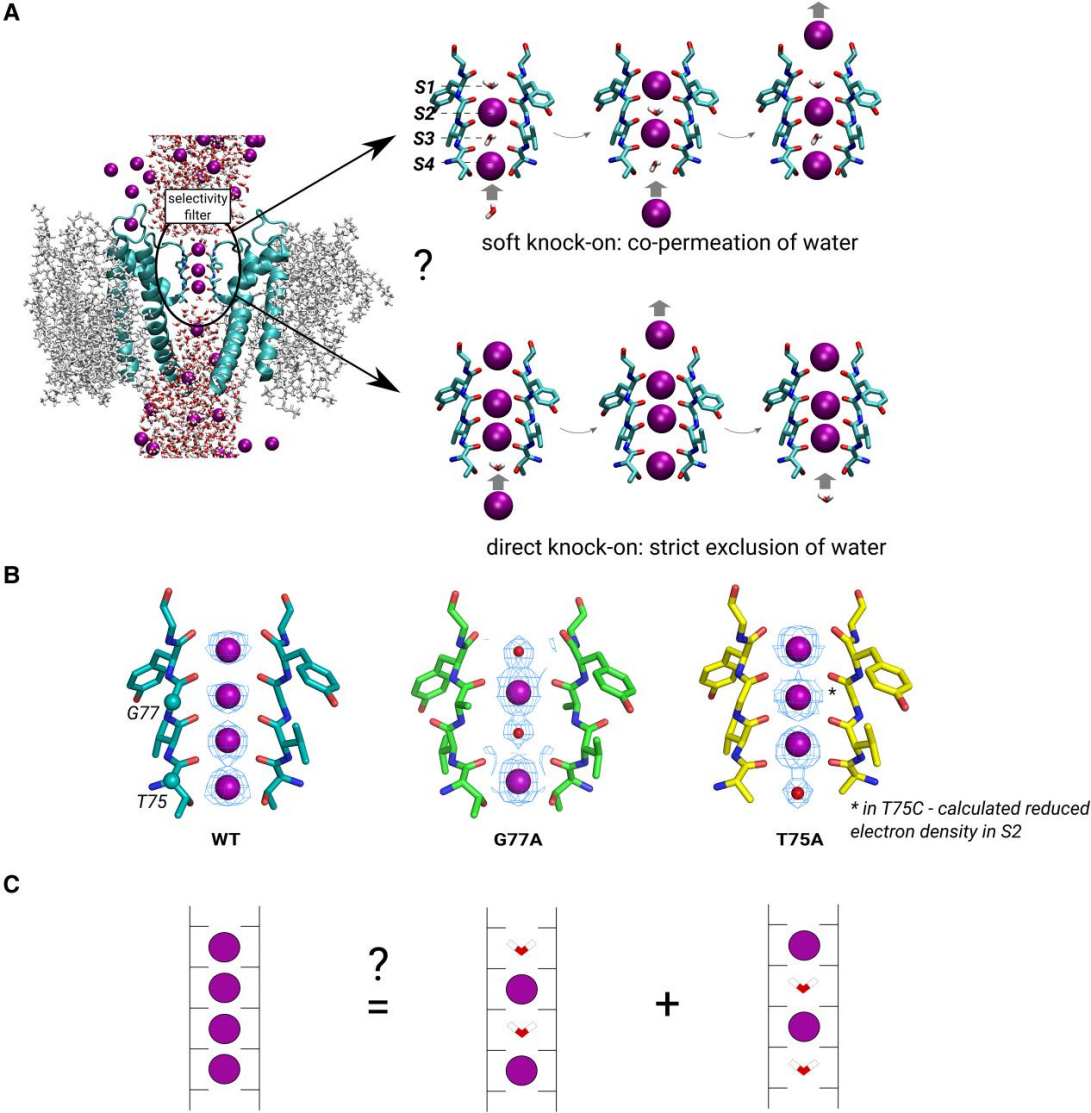
Overview of ion permeation mechanisms in potassium channels. A) Side view of a structure of KcsA embedded in a lipid membrane, with the highly conserved SF highlighted. K^+^ ions are shown as purple spheres. On the right, main K^+^/water SF configurations that occur in the two proposed permeation mechanisms, soft and direct knock-on, are shown. B) SF configurations observed in wild type (WT) (PDB ID 5vk6), G77A (PDB ID 6nfu), and T75A (PDB ID 6by3) KcsA. C) One possible interpretation of the fully K^+^-occupied SF observed in structures of many WT potassium channels.

K^+^ channels possess a highly conserved selectivity filter (SF), usually with a signature sequence TVGYG (5, 6). The SF is the narrowest part of a K^+^ channel pore and serves as the functional core of the channel. It consists of 4 K^+^ binding sites—S1 to S4, respectively—lined by backbone carbonyls (S1–S4) and threonine hydroxyls (S4 only), pointing toward the permeation pathway (Fig. 1A). The K^+^ permeation mechanism in K^+^ channels is defined by specific arrangements of K^+^ ions and, possibly, water molecules inside SF, occurring during ion translocation through the channel. Currently, there are two primary models of K^+^ permeation in K^+^ channels: the “soft knock-on” and “direct knock-on” mechanisms (4). Soft knock-on was proposed following the first structural data: it states that the SF is occupied by 2 K^+^ simultaneously—either in S1 and S3, or in S2 and S4—and by water molecules in the remaining two sites (7, 8). This separation of two K^+^ ions by a water molecule was included to prevent seemingly too strong electrostatic repulsion between K^+^ ions, if they would occupy neighboring sites. Accordingly, in the course of a permeation event, given an initial configuration “KWKW” (K stands for a K^+^ ion and W for a water molecule, in sites S1 to S4 respectively), an ion approaching S4 from the intracellular side “knocks on” the contents of the SF to a configuration “WKWK” (Fig. 1A), and vice versa—thus, for each K^+^ ion there is one water molecule permeating the channel. A very different process takes place in the direct knock-on mechanism, as water does not permeate the channel. Rather, K^+^ ions are completely dehydrated in the SF, and direct ion–ion contacts are formed (Fig. 1A) (9). This complete dehydration of K^+^ ions in the SF makes ion permeation inherently selective toward K^+^ compared to Na^+^ due to the lower dehydration free energy of the former (10). In addition, the strong electrostatic repulsion arising from the direct ion–ion contacts was proposed to drive fast permeation in K^+^ channels (9). Importantly, both soft and direct knock-on mechanisms follow a strict single-file ion (and water) permeation and an unperturbed SF conformation is assumed during ion permeation.

In extensive experimental and computational studies of K^+^ permeation mechanisms, some results support soft knock-on [e.g. interpretations of crystal structures (7, 11), streaming potentials (12–14), or global fits by electrophysiology (15)], whereas others favor direct knock-on [e.g. SF occupied by 3 to 4 K^+^ in anomalous diffraction data (16–18) or “KKKK” configuration (Fig. 1B), computational electrophysiology molecular dynamics (MD) simulations, and free energy calculations using polarizable force fields (9, 10, 19) or solid-state NMR measurements (4, 20, 21)]. Thus, the debate on which mechanism actually takes place in K^+^ channels remains unresolved. Within this debate, the data on SF mutants: G77A and T75X (*X* = A, C, G) in the model K^+^ channel KcsA, present an intriguing case. The G77A mutation effectively removes the S3 binding site, as the backbone carbonyls of V76 point away from the ion pathway (carbonyl “flip”) in the crystal structure (Fig. 1B) (22). Interestingly, the structure shows both S3 and S1 occupied not by K^+^, but by what has been interpreted as water molecules instead, or the “WKWK” configuration [however, it should be noted that without anomalous diffraction data, it is difficult to tell whether this density corresponds to water molecules or e.g. reduced K^+^ occupancy (17)]. As mentioned above, one of the arguments in favor of direct knock-on is the “KKKK” configuration in crystal structures of most wild type (WT) K^+^ channels. However, an alternative interpretation posits the “KKKK” configuration as a superposition of soft knock-on “WKWK” and “KWKW” configurations (8) (Fig. 1C). In this context, the structure of G77A was originally interpreted as one isolating the “WKWK” configuration, the same configuration that would occur during permeation in WT via the soft knock-on mechanism (22). In the same spirit, T75C and T75G mutations not only remove the S4 binding site, but lower the K^+^ occupancy at S2, which has been interpreted as an isolated “KWKW” configuration (Fig. 1B) (23, 24). Interestingly, the occurrence of soft knock-on-specific configurations in G77A and T75X mutants has been taken as evidence for the soft knock-on mechanism in WT K^+^ channels (22). Functionally, mutations at both 75 and 77 positions largely decrease K^+^ conductance [ca. 32-fold for G77A (22) and between 3- and 17-fold for T75X (23, 25)]. The data on ion selectivity are ambiguous: liposome flux assay (LFA) experiments showed WT-like K^+^/Na^+^ selectivity in KcsA G77A (22), whereas no selectivity measurements have been conducted (to the best of our knowledge) on T75X mutants. For other channels with the conserved SF sequence (TVGYG), the electrophysiological selectivity measurements are conflicting: hKv1.5 T480A and Shaker T442A (25) retained K^+^ selectivity, while NaK2K T63A and MthK T59A were nonselective (26).

In this work, we study ion permeation mechanisms in the G77A and T75A mutants of KcsA using MD simulations with applied voltage with two modern fixed-charge force fields. In our simulations, WT KcsA permeates strictly via water-free direct knock-on, whereas both mutations drastically affect the SF conformational dynamics, coupled with permeation mechanisms incompatible with direct knock-on. In fact, the strict water-free K^+^ permeation is compromised in G77A and T75A, leading to largely reduced permeation rates, in good agreement with experimental data (22, 23, 25). The K^+^/Na^+^ selectivity of the mutants is similarly diminished. Our findings not only present a case when the structural and mutagenesis data should be interpreted with caution in the context of ion permeation mechanisms, but also support the idea that exclusion of water from the SF is necessary for the hallmark high conductance/high selectivity combination in K^+^ channels.

## Results

### Ion permeation in the WT channel

First, we simulated ion conduction in WT KcsA at the membrane voltage of 300 mV, using the CHARMM36m (27) and Amber14sb (28) force fields. In simulations with CHARMM36m, we observed 40 K^+^ permeation events within the cumulative 10-μs simulation time, the majority of which occurred via direct knock-on. In 5 out of 10 simulation replicas, a water molecule entered the central parts of the SF (S2/S3). In these cases, further ion permeation was halted (Fig. S1A), suggesting that water in the SF transiently blocks ion permeation. In one simulation replica, the SF underwent drastic conformational changes with a large increase in diameter at S3, S2, and S1 that allowed permeation of both K^+^ and water (Fig. S1B). In contrast, in Amber14sb, the SF was stable and inaccessible to water (Fig. S1C), allowing to observe 81 permeation events that occurred exclusively via direct knock-on. The calculated outward conductances are therefore 2.1 ± 1.1 pS and 4.3 ± 1.1 pS in CHARMM36m and Amber14sb, respectively, an order of magnitude lower than the experimental values for KcsA (3)—a known effect of K^+^ channel simulations using fixed-charge models (4).

The SF of WT KcsA in MD simulations with the CHARMM36 force field can transition toward the “constricted” conformation, related to C-type inactivation, much faster than in experiments—within hundreds of nanoseconds instead of seconds (29–31). While we observed instabilities in the SF in certain simulation replicas, such transitions did not occur in our simulations (Fig. S2), possibly due to a lower temperature in our study, or limited sampling. We noticed however, that the SF was on average narrower in CHARMM36m compared to Amber14sb at G77 (by 0.048 ± 0.005 nm, distance between CA atoms of opposing subunits) and G79 (0.024 ± 0.005 nm) (Fig. S3A). Our previous work on MthK channels showed that subtle changes in the SF diameter can have a major effect on outward currents (32); thus, it is feasible that such an effect might be present in KcsA as well.

Finally, we tested the selectivity of WT KcsA by simulating in the presence of Na^+^ ions (instead of K^+^). To note, selectivity is often used to describe ion permeation in conditions when two ion species are present simultaneously (biionic conditions); in this study, however, we will use the term “selectivity” for both single-ion and biionic conditions. We did not observe any Na^+^ permeation in WT KcsA using CHARMM36m and only two permeation events in Amber14sb (PNa^+^/PK^+^ = 0.03 ± 0.05), showing strict K^+^ selectivity, in agreement with experiments (3).

### Ion permeation in KcsA E71A

As the WT KcsA showed unsustained K^+^ permeation frequently blocked by water and overall low conductance, we investigated its noninactivating E71A mutant (Fig. S4A), to obtain a clearer picture of K^+^ permeation, since earlier MD simulations as well as experiments showed higher currents and stability of KcsA E71A compared to WT (30, 33). Similarly, in our simulations, KcsA E71A showed sustained currents in both force fields. K^+^ permeation occurred consistently via direct knock-on, with rare events of water molecules entering the SF in CHARMM36m that were, however, able to leave so that water-free permeation continued (Fig. S4B). In total, we observed 494 permeation events over the cumulative 10 μs of simulations with KcsA E71A in CHARMM36m, and 124 in Amber14sb (Table S1)—corresponding to 26.4 ± 4.3 pS and 6.6 ± 1.4 pS. The increased conductance of E71A correlated with the SF width: E71A is 0.05 ± 0.02 nm wider at G79 compared to WT (CHARMM36m, Fig. S3B), in line with our previous work on MthK and its KcsA-like V55E mutant (32).

At the same time, KcsA E71A retained the strict K^+^ selectivity: no Na^+^ permeation was observed in simulations with the CHARMM36m force field and only three Na^+^ permeation events in Amber14sb (PNa^+^/PK^+^ = 0.02 ± 0.04). It should be noted that the experimental measurements of E71A K^+^/Na^+^ selectivity are not fully clear: while ^22^Na^+^ flux measurements of E71A suggested lower selectivity compared to WT (34), reversal potential shifts showed no significant change in selectivity in E71A (34, 35). A possible explanation for this observation is the propensity of the E71A SF to adopt a specific conformation in pure Na^+^ conditions [PDB ID 3OGC (34), although a similar conformation has been observed in K^+^-containing solution as well, PDB ID 2ATK (35)], not seen in WT KcsA (as it inactivates instead). This E71A-specific conformation shows flipped V76 carbonyls and flipped side chains of the residue D80. In fact, we observed rare hints of such conformation in our simulations of E71A in pure Na^+^ (Fig. S5), although likely due to the limited sampling and therefore short simulated times with this conformation, we did not record any Na^+^ permeation events in it. Therefore, we believe we can safely assume that, for this study, WT KcsA and E71A KcsA display similar K^+^/Na^+^ selectivity, as long as their SFs retain the canonical, conductive conformation.

To summarize, in our simulations KcsA E71A showed essentially the same permeation mechanism (direct knock-on) and ion selectivity as the WT channel, albeit with much higher currents. We thus conducted our study in the following manner: first, we introduced the SF mutations into KcsA E71A, and investigated their effect on K^+^ permeation in G77A/E71A and T75A/E71A constructs. As control systems, we simulated the single G77A and T75A mutants and compared their behavior to the “pure” WT KcsA ( that is, without the E71A mutation), to account for possible biases of channel sequences and force fields, and get the comprehensive picture of the effect of SF mutations on ion permeation.

### Effect of the G77A mutation

**Fig. 2.**
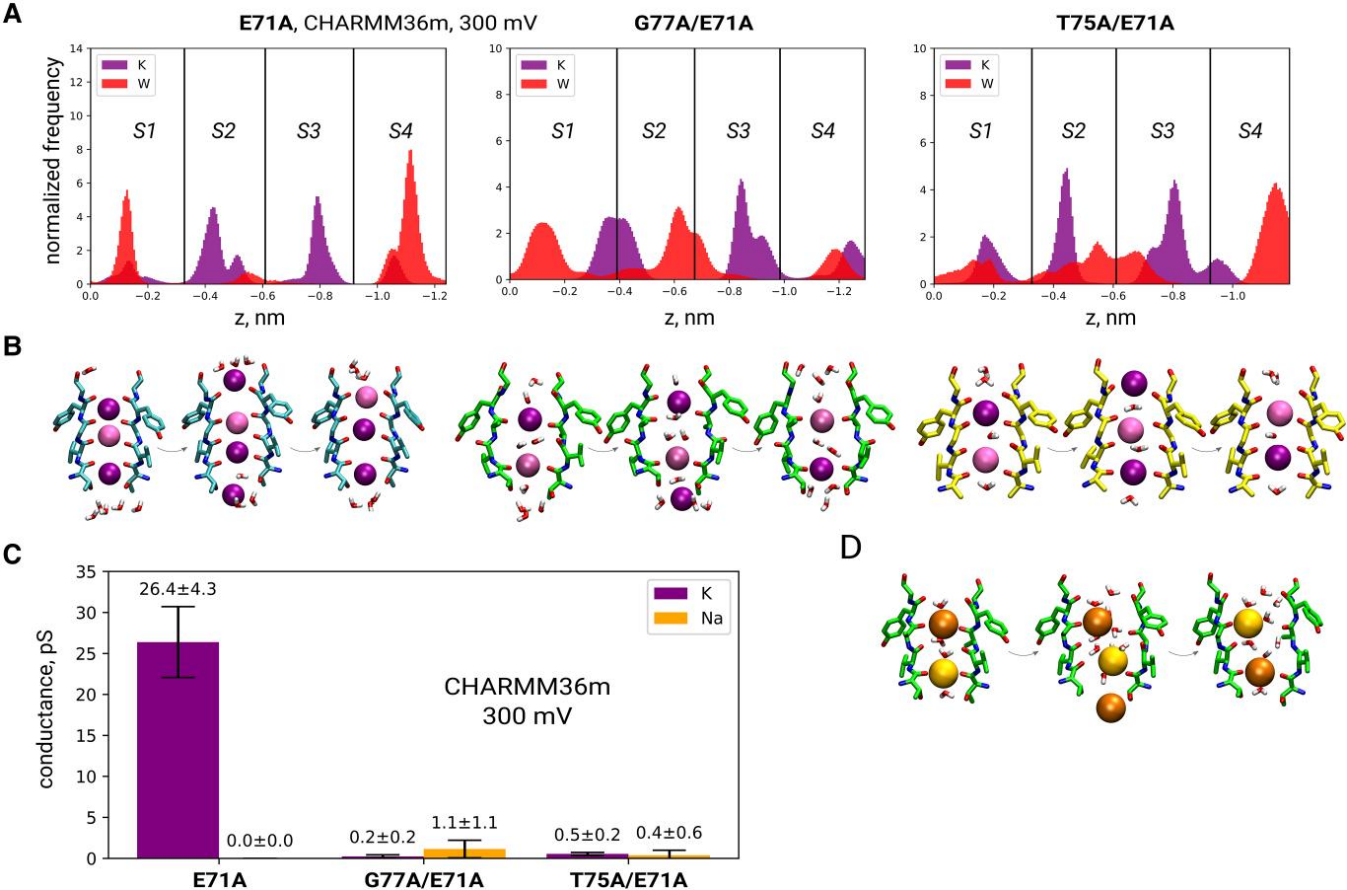
A) K^+^/water distributions along the *z*-axis in SF of KcsA E71A, G77A/E71A, and T75A/E71A from all simulation replicas in CHARMM36m at 300 mV, with B) respective representative K^+^/water configurations corresponding to a single K^+^ permeation event. Shift to a mechanism involving co-permeation of K^+^ and water is observed upon introduction of G77A and T75A mutations. C) Mutations reduce the K^+^ conductance and impair selectivity against Na^+^ (CHARMM36m, 300 mV), with D) showing the representative SF configurations observed in a Na^+^ permeation event in KcsA G77A/E71A.

**Fig. 3.**
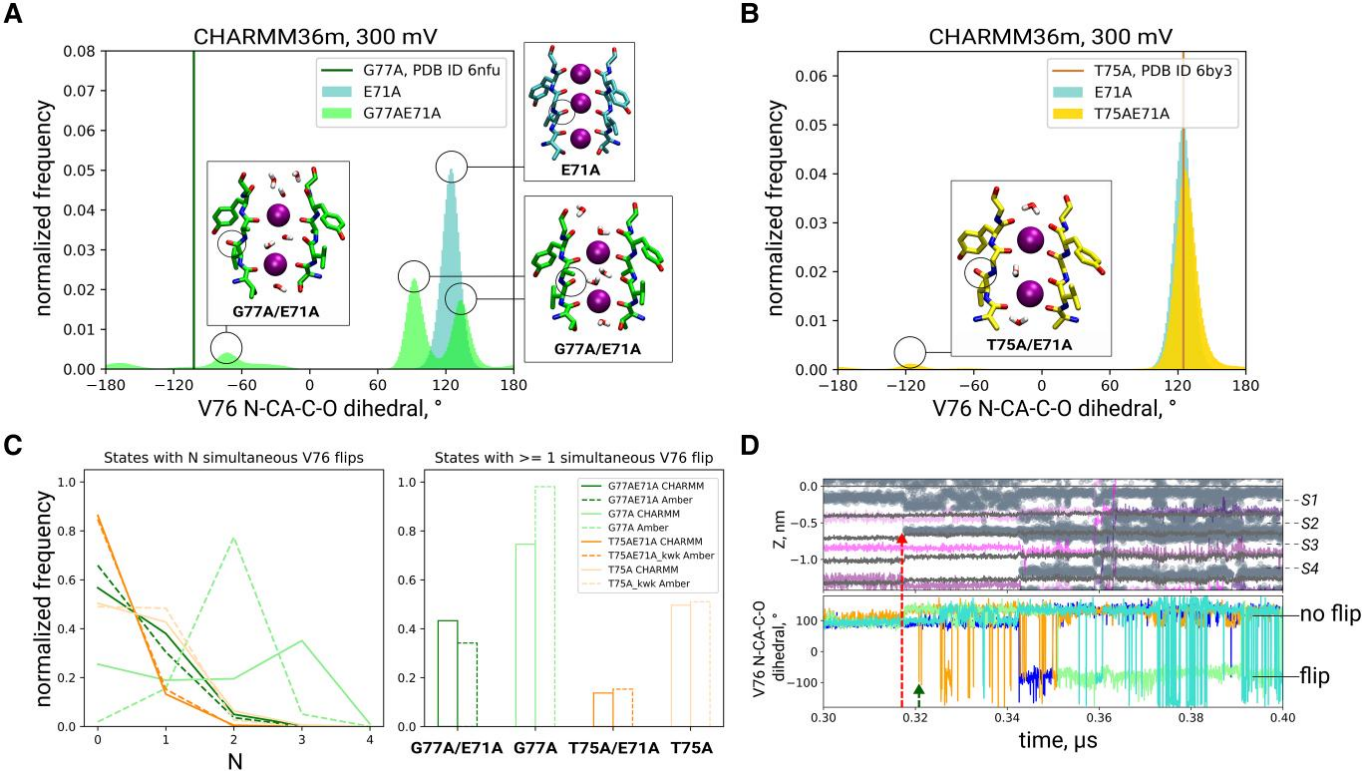
SF mutants had a higher frequency of V76 carbonyl flipping, represented by V76 N–CA–C–O dihedral angle distributions for A) E71A and G77A/E71A KcsA, with the dihedral angle in the G77A KcsA crystal structure indicated (PDB ID 6nfu), and B) E71A and T75A/E71A KcsA, similarly indicating the dihedral angle in the T75A KcsA crystal structure (PDB ID 6by3). C) Fraction of states with various numbers (N) of simultaneously flipped carbonyls (left), and the fraction of states where at least one V76 carbonyl is flipped (right). In T75A and T75A/E71A, in Amber14sb flipping has been detected only when a soft knock-on-like starting SF configuration was used (“kwk”). D) Time traces of K^+^ ions (purple lines) and water molecules (gray scatter), oxygens of SF backbone carbonyls and of threonine hydroxyls (gray lines) (up), aligned with time traces of V76 N–CA–C–O dihedral for G77A/E71A, illustrate how entrance of water into S2/S3 (red arrow) promotes V76 carbonyl flipping (green arrow).

The introduction of G77A into KcsA E71A led to a drastic change in the permeation mechanism: the mutated channel co-permeated water molecules, thus compromising the water-free direct knock-on mechanism (Fig. 2A, B, *middle*). Although this mechanism resembled soft knock-on, there were some differences—K^+^ ions permeated by jumping directly from the KW**K**W configuration to **K**WKW—without the intermediate W**K**WK that is expected according to the canonical definition of the soft knock-on mechanism (Fig. S6A). Additionally, the number of intervening water molecules between K^+^ ions was often 2 or more (instead of expected 1 water molecule). The dominance of the KWKW occupancy pattern is not compatible with electron densities in the SF of WT channels that correspond to similar ion occupancy for all four central ion binding sites (6, 11, 17), hence rendering this permeation mechanism unlikely for the WT channel. Importantly, the average position of K^+^ and water inside the SF in our simulations is in good agreement with the crystal structure of G77A (22) (Fig. 2A), with an exception of S4. In the crystal structure, S4 is occupied by a K^+^ ion, whereas simulations show a larger K^+^ density at S3 in both force fields (Figs. 2A and S7A). This discrepancy may be related to V76 carbonyl flipping dynamics: in the crystal structure, all 4 V76 are flipped, while in our simulations the number of simultaneously flipped V76 ranged between 0 and 4, and conformations with a larger number of simultaneous flips were less frequent (Fig. 3C). Accordingly, the fraction of frames where K^+^ occupied S4 instead of S3 increased with the number of simultaneously flipped V76 (Fig. S7B).

The above-mentioned shift in the ion permeation mechanism was accompanied by a drastic reduction in K^+^ conductance for G77A/E71A compared to E71A. In total, we observed only 16 K^+^ permeation events in CHARMM36m and 4 in Amber14sb, over ∼30-μs and 10-μs total simulation time, respectively (Table S1), corresponding to an average ∼100-fold (CHARMM36m) and ∼30-fold (Amber14sb) reduction in outward conductance (Figs. 2C and S8A) due to the mutation. This decrease agrees well with experimental estimates (32-fold reduction) (22). Further, K^+^/Na+ ion selectivity of G77A/E71A was clearly impaired in CHARMM36m, as the channel permeated Na^+^ ions at a rate similar to K^+^ (Fig. 2C). Na^+^ permeation, akin to K^+^, featured water co-permeation (Figs. 2D, S6B). While the loss of ion selectivity has a straightforward explanation within the direct knock-on framework (10) (lack of complete ion desolvation reduces K^+^ vs Na^+^ selectivity, due to differences in solvation free energies), it is in apparent contrast to the interpretation of the LFA experiments in which the G77A mutant does show ion selectivity (22). We address these discrepancies in the Discussion. To get a more complete comparison to the LFA measurements, where the channel is oriented randomly in the liposomal membrane, we also simulated inward permeation (at −300 mV) in CHARMM36m. Similar to simulations at positive voltage, G77A/E71A was equally permeable to Na^+^ and K^+^, while E71A retained its selectivity (Fig. S8B). In contrast, we did not observe any Na^+^ permeation in simulations in Amber14sb. Given the very low K^+^ permeation rate of G77A/E71A with this force field (four events in 10 μs), it is difficult to conclude whether this mutant is actually selective in this force field. Finally, we tested biionic conditions with two K^+^/Na^+^ ratios (2:1 and 1:2) (Table S1); however, we saw little to no permeation of either ion in both CHARMM36m and Amber14sb at +300 mV and some permeation of both ions in CHARMM36m at −300 mV (Fig. S8C). Control simulations of KcsA G77A without the E71A mutation also showed a water-mediated permeation mechanism, with a reduced average outward conductance compared to WT in CHARMM36m (∼2-fold) (Fig. S9A). Ion selectivity was compromised in CHARMM36m at both positive and negative voltages, as well as in biionic conditions (tested at +300 mV) (Fig. S9B–D). In Amber14sb, we did not observe K^+^ or Na^+^ permeation, whereas flipping of V76 occurred in both force fields (Fig. 3C).

### Effect of the T75A mutation

T75A removes the hydroxyl groups that form the bottom of the S4 ion binding site. Consequently, in the crystal structure of KcsA T75A the K^+^ ion in S4 is replaced by a water molecule (25) (Fig. 1B, *right*). In our simulations of KcsA T75A/E71A, this had a dramatic effect on K^+^ permeation, in a force field-dependent manner. In Amber14sb, we did not observe any ion permeation, regardless of whether the initial SF configuration was direct or soft knock-on-like (Table S1). In contrast, in CHARMM36m, the introduction of the T75A mutation led to co-permeation of K^+^ and water (Figs. 2A, *right*, S10). Interestingly, while in the crystal structure of KcsA T75A the SF carbonyls are not flipped, we observed a tendency of T75A/E71A to flip at V76, in a similar manner to G77A/E71A and G77A (Fig. 3A–C). Flipping correlated with water entry into the SF, suggesting that it could be a general feature of water-filled SFs (Figs. 3D, S12C).

KcsA T75A/E71A in CHARMM36m showed a low outward K^+^ conductance of 0.5 ± 0.2 pS (Fig. 2C) (10 permeation events in 10 μs), which is in good agreement with experiments (25). Further, the mutant was equally permeable to K^+^ and Na^+^ (Fig. 2C), suggesting compromised ion selectivity. We cannot compare this prediction with experiments, as ion selectivity measurements are lacking for KcsA T75X constructs, and the data for other K^+^ channels are inconclusive (see Introduction).

In simulations of KcsA T75A without the E71A mutation, we also observed low K^+^ conductance of 0.40 ± 0.16 pS (15 K^+^ permeation events over 20 μs) in CHARMM36m (Fig. S11A) and no ion permeation in Amber14sb. The permeation mechanism was generally similar to that of T75A/E71A, featuring co-permeation of water and K^+^. Interestingly, while the double mutant showed a preference for a stepwise motion of the KWK pair through the SF (Fig. S10), here, direct jumps from KW**K**W to **K**WKW occurred, without the intermediate configuration W**K**WK (Fig. S11C). Again, this is at odds with the original soft knock-on mechanism that requires KWKW and WKWK to occur with similar probabilities, in order to explain the similar electron densities at the four central ion binding sites in the SF of nonmutated channels. Accordingly, the resulting K^+^ density profile displays a relatively diminished K^+^ density at S2 compared to the double mutant (Fig. S15), reminiscent of the crystal structure of KcsA T75C and T75G (23, 24). The frequency of V76 flipping was higher compared to the double mutant (Fig. 3C), possibly explaining the reduced K^+^ density at S2 by perturbed K^+^–V76 carbonyl interactions. Regarding ion selectivity, 2 Na^+^ permeation events in single-ion conditions over the total 25 μs of simulation were detected (conductance of 0.05 ± 0.08 pS).

As noted before, the starting structures for all simulations mentioned were prepared by introducing the appropriate mutations into the KcsA E71A crystal structure. However, a crystal structure of KcsA T75A in the open state is available (25). We thus built and performed simulations of this KcsA T75A structure, together with WT KcsA back-mutated from the same structure (CHARMM36m, +300 mV). The results were in broad accord with our main simulation set, with permeation via direct knock-on in WT KcsA, with occasional water occupancy in the SF, low conductance but K^+^/Na^+^ selectivity, and soft knock-on with low conductance in T75A. Interestingly, while the preference for **K**WKW to KW**K**W jumps was not as pronounced compared to T75A from the main simulation set, the relative K^+^ density drop in S2 was nevertheless present (Fig. S16). Additionally, in this new system we could detect a higher number of both K^+^ and Na^+^ permeation events and conclude lower selectivity in T75A with higher confidence (Fig. S11B).

## Discussion

In this work, we studied the effect of the G77A and T75A mutations on the ion permeation mechanism and selectivity in the model potassium channel KcsA channel using MD simulations with applied voltage, using two popular force fields. We show that the introduction of these mutations leads to a complete change in the K^+^ permeation mechanism—from a water-free direct knock-on to mechanisms that feature co-permeation of water. This, in turn, dramatically reduces ion permeation rates, in good agreement with experimental data. Importantly, we introduced mutations into the SFs of both WT KcsA and its noninactivating E71A variant; this suggests that the observation of the shift toward water-mediated permeation mechanisms and low conductance is solely due to the introduced mutations and independent of the initial channel.

The shift in the permeation mechanism is rationalized by the altered conformational landscape of mutated SFs, reminiscing our previous work on selective and nonselective permeation in K^+^ channels and their nonselective counterparts, as well as single-molecule Förster resonance energy transfer (FRET) experiments from the Nichols lab. Indeed, we previously observed dilated and more dynamic SFs of nonselective channels that allow simultaneous permeation of ions and water molecules, and we linked this increased water co-permeation to reduced K^+^/Na^+^ selectivity (10). Of particular interest, a series of FRET experiments on KirBac1.1 (36), NaK2K, and TREK-2 (37) channels showed similar, ion-dependent SF conformational states in all of these channels. In these experiments, the presence of K^+^ in the SF promotes a compact, Rb^+^-permeable conformation of the SF, whereas in Na^+^ the SF adopts a dilated, Na^+^, and water-permeable state, thus offering an explanation how water-free direct knock-on can occur via compact states, and yet still display water permeation through Na^+^-induced dilated conformations. Another study on S3 glycine mutants in KirBac1.1 also showed more dynamic SFs compared to WT and consequently reduced K^+^/Na^+^ selectivity (38). In simulations presented in this work, the main source of increased SF dynamics is associated with V76 carbonyls. In the crystal structure of KcsA G77A, S2- and S3-forming V76 carbonyls are in the flipped state as they point away from the pore axis (22) (Fig. 1B*, middle*). This V76 flipping occurs spontaneously in our simulations of G77A/E71A (and G77A). In contrast to the crystal structure, however, not all 4 V76 are simultaneously flipped throughout the simulations, but rather the number of flipped carbonyls fluctuates between 0 and 4. The details of carbonyl dynamics vary between the systems (with or without the E71A mutation) and the force field used (Fig. 3C). These differences may be explained by the fact that the crystal structure was solved with an imposed 4-fold symmetry and also at a lower temperature (100 K) than the one we used in our simulations (290 K). Consequently, low energetic barriers between flipped/nonflipped states are crossed. Notably, this carbonyl dynamics directly affects the position of the lower ion in the SF, as it is more often located in S4 (i.e. its crystallographic site) when more V76 are simultaneously flipped (Fig. S7B).

Unexpectedly, we observed a similar behavior in the T75A mutant. Even though it only removes the hydroxyl groups of the S4-forming T75 residue and otherwise does not affect the SF conformation in the crystal structure (Fig. 1B, *right*) (25), we observed flipping of V76 carbonyls in simulations of T75A as well. A unifying factor was a switch (either transient or not) to a water-mediated permeation mechanism; indeed, for all systems we studied, we found a clear correlation between V76 flipping and water entrance into the K^+^ binding sites formed by V76, S2, and S3, including WT KcsA and E71A that showed some frequency of flipping too (Fig. S12A, B). Similar correlation between water presence in the SF and flipping of S2/S3 carbonyls was demonstrated in previous MD studies of MthK (39), TREK-2 (40), and HERG (41) channels. We analyzed the causal relationship of flips and other factors; the vast majority of flipping events happened when water was already in S2/S3 for most systems, despite a sizable fraction of frames (CHARMM36m) with no water in S2/S3, thus further supporting the preference of flipped states for water presence in the SF (Fig. S12C). G77A in CHARMM36m was a notable exception, with more than 30% of flips starting with no water in S2/S3, indicating also the role of intrinsic propensity to flip of a given channel. Interestingly, however, flipping events that started with water in S2/S3 tended to last longer in all systems (Fig. S12D). While we previously described the ion occupancy of lower sites (S3/S4) correlating with the number of simultaneous V76 flips, we did not find the absence of ions in S2/S3 to be necessary for flips to occur (Fig. S13).

A plausible structural explanation for the effect of water on flips could be in the several types of hydrogen bond networks that water molecules formed with carbonyl oxygens. First, water interacted with V76 carbonyls while inside the SF, thus it could potentially stabilize some and destabilize other flipping configurations (Fig. S14A). Second, water behind the SF (primarily in G77A/E71A and T75A/E71A, where bulky glutamates behind the SF were removed) could sometimes interact with and stabilize the flipped V76 (Fig. S14B). Taken together, however, carbonyl flipping is likely affected by a combination of factors, such as the presence and configuration of water molecules in and around the SF, potentially ion configuration, as well as the intrinsic propensity to flip of specific SF sequences and the regions behind it (Fig. 3C).

The exquisite ion selectivity of K^+^ channels arises from a balance of interactions between ions, water, and the SF. We have previously shown that the direct knock-on mechanism is intrinsically selective for K^+^ against Na^+^, due to the difference in ion dehydration free energies (10); therefore, we expected a diminished ion selectivity for the water-permeating mutants with dilated SFs. In line with these expectations, mutants of other K^+^ channels at the second glycine in the SF (corresponding to G77 in KcsA), i.e. BK G354S (42), GIRK2 G156S (human G154S) (43, 44), and Shaker G376A (5), show a loss of ion selectivity in electrophysiology. On the contrary, LFA measurements of KcsA G77A suggested that it conserves ion selectivity, and isothermal titration calorimetry (ITC) showed similar binding affinities of ions to the WT and mutant channels (22). In our simulations, both G77A and G77A/E71A are clearly nonselective in CHARMM36m, at both positive and negative voltages. G77A in CHARMM36m, where we were able to detect ion permeation when both K^+^ and Na^+^ were present, was nonselective in these conditions as well. In Amber14sb, these mutants did not display any Na^+^ permeation, but also K^+^ conductance was very low (i.e. only four permeation events in 10 μs for G77A/E71A, and G77A was nonconductive), which prevented us from making conclusions about their ion selectivity with this force field. One possible reason for this discrepancy between LFA experiments and MD simulations could be the different driving forces of ion permeation: while an applied electric potential was used in our study, in LFA ions are driven in or out of liposomes by their concentration gradients. This can lead to uncertainty in the instantaneous transmembrane voltage. Second, the intracellular cavity of KcsA was shown to be blocked by Na^+^ in a voltage-dependent manner (45). Na^+^ block can be relieved either by diffusion of Na^+^ back into the intracellular solution, or via a “punchthrough” mechanism through the SF by incoming ions; however, it is not clear to what extent this phenomenon is present for KcsA in LFA and how it would affect the results. Then, the method showed an overall higher current for K^+^ compared to Na^+^ in this setup, but did not preclude Na^+^ currents, nor quantify the effect of G77A on ion selectivity. In this vein, a possible interpretation of the LFA results that could be reconciled with our MD data is that even if KcsA G77A possesses some preference for K^+^ against Na^+^, the selectivity is still diminished compared to WT, as it is the case for the mutants of the glycine of BK (PNa^+^/PK^+^ ∼0.2) (42), GIRK (∼0.8) (43, 44), and Shaker (∼0.8) (5) channels. Finally, it is also not straightforward to interpret the similar binding affinities in ITC, as the number of binding sites of K^+^ in the channel in ITC is not unequivocal (as well as whether the same site is titrated in both WT and G77A), but also ITC does not provide energetic barriers crucial for ion permeability.

Notably, mutations of the S3 glycine in K^+^ channels in humans are associated with disorders such as progressive cerebellar ataxia [BK G354S (42)] and Keppen–Lubinsky syndrome [GIRK2 G154S (46)]. BK and GIRK2 are expressed in multiple tissues in humans and participate in many processes including control of the smooth muscle tone and contributing to the resting potential of neurons, respectively (47, 48). As mentioned above, these mutants have diminished conductance and ion selectivity, and in a previous MD study of GIRK2 G154S, water-mediated K^+^ permeation was reported (49). Taken together with our results, it is plausible that a shift in the ion permeation mechanism happens in the mutants of these channels as well, disrupting proper ion permeation and leading to these disease phenotypes. This could provide a mechanistic basis that may guide future development of drugs targeting those disorders.

The T75A mutation behaved similarly to G77A in our simulations: it displayed reduced selectivity for K^+^ (for both single and double mutations) in CHARMM36m at positive voltage (we did not observe any K^+^ or Na^+^ permeation for either system at negative voltage or in Amber14sb), with identical K^+^ and Na^+^ conductances in T75A/E71A, and somewhat higher conductance for K^+^ in T75A (Figs. 2C, S11A, B). As mentioned, we are not aware of ion selectivity measurements for KcsA T75A. However, the ion selectivity measurements of other K^+^ channels with mutated threonine at the same location are conflicting: Kv1.5 T480A and Shaker T442A mutants were selective (25), while NaK2K T63A and MthK T59A were nonselective (26). This channel-dependent effect of the SF threonine substitution can indicate channel-dependent shifts in the ion permeation mechanism, posing an intriguing question—do selective mutants conserve the WT permeation mechanism, while nonselective ones have it switched to a non-WT mechanism, similarly to what we observed for KcsA T75A?

**Fig. 4.**
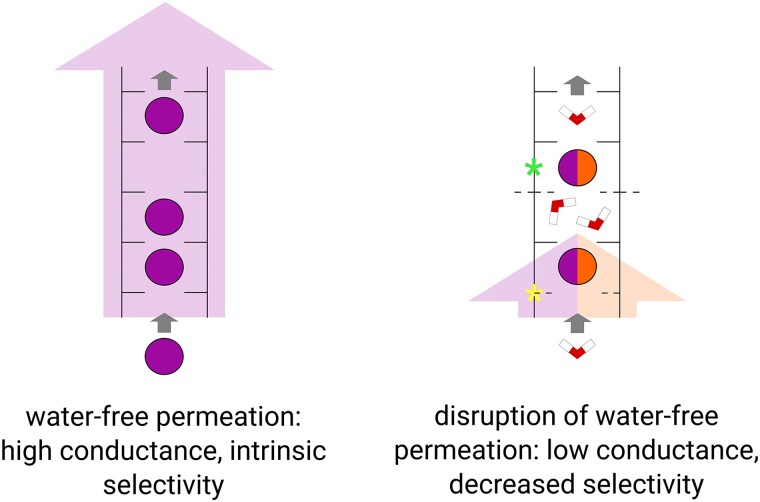
Schematic view of ion permeation in WT K^+^ channels (left) and the SF mutants (right) affecting either S4 (yellow) or S2 (green) K^+^ binding sites. While K^+^ permeation via direct knock-on is characterized by high conductance and high selectivity for K^+^ ions compared to Na^+^, disrupting the water-free permeation by e.g. introducing mutations to the SF impedes both permeation rates and ion selectivity. Both mutations we studied promoted flipping of V76 carbonyls (right, dashed lines in the middle), regardless of whether they affected the adjacent G77 or distant T75, which we also link to the destabilizing effect of water presence in the SF. Note that the SF configurations on the figure serve as generic representations of direct and soft knock-on mechanisms; in our simulations, a variety of K^+^ and/or water configurations is observed for each mechanism.

Recently, the fact that crystal structures of G77A and T75X mutations of KcsA show ion/water configurations of their SFs has been interpreted as evidence for the soft knock-on mechanism in WT channels as well (22). In this context, the “KKKK” configuration found in SFs of WT channels is viewed as a superposition of “WKWK” and “KWKW” configurations (characteristic for soft knock-on, Fig. 1A). Consequently, KcsA G77A has been proposed to isolate “WKWK”, whereas KcsA T75X would isolate “KWKW”, as T75X showed a decreased K^+^ density at S2 (on top of eliminating S4). This presents an argument in favor of soft knock-on in WT KcsA, under a critical assumption that the permeation mechanism is not affected by the introduced mutations. Our results, however, show that both mutations largely perturb the conformational landscape of the SF, to an extent that makes the permeation mechanism in the mutants incompatible with that of the WT. Thus, we argue the experimental data on KcsA G77A and T75X do not contradict the direct knock-on mechanism in WT K^+^ channels. Instead, G77A and T75X simply conduct ions (and water) via an entirely different mechanism. Water-free K^+^ permeation within the direct knock-on framework as a prerequisite for combined high conductance and selectivity of K^+^ channels is supported by several studies, both computational and experimental (4), and our current results fit very well within this framework, thus further solidifying direct knock-on as the permeation mechanism in K^+^ channels (Fig. 4).

## Materials and methods

### System preparation

The starting structure for the KcsA noninactivating mutant E71A in the open conformation [PDB ID: 5vk6 (50)], embedded in a lipid bilayer, was built using CHARMM-GUI (51, 52). Its sequence (residues 26 to 121, preserving the E71A mutation) was mutated to match UniProtKB entry P0A334. N- and C-termini were acetylated and methylamidated, respectively. The system contained 39 1,2-dioleoyl-sn-glycero-3-phospho-(1′-rac-glycerol) (DOPG) and 105 1,2-dioleoyl-sn-glycero-3-phosphoethanolamine (DOPE) molecules in the lipid bilayer; 11,257 water molecules; 163 K^+^ and 140 Cl^−^ ions to yield a neutral system; and a K^+^ concentration of 0.8 M in the water phase. Four of DOPG molecules were placed in their respective crystallographic binding sites in KcsA. E118 and E120 were protonated to stabilize the open state of the channel (53).

The KcsA T75A/E71A system was built by manually introducing a corresponding mutation to the KcsA E71A system (4 × KcsA monomers). To obtain the WT structure, A71 in the KcsA E71A system was reverted to a glutamate which was then protonated in line with ssNMR results (54). As the KcsA E71A structure has 2 water molecules per monomer that establish the hydrogen bonding network behind the SF, compared to 1 in WT (6), the additional water molecules were removed accordingly. SF mutants without E71A were obtained by manually introducing G77A or T75A to this WT structure. KcsA G77A/E71A was built separately in CHARMM-GUI from E71A 5vk6 using a similar protocol and contained, in addition to the protein 33 DOPG and 87 DOPE molecules, 9,315 water molecules, 134 K^+^ and 117 Cl^−^, resulting in an electrically neutral system with the 0.8 M concentration of K^+^ in the water phase.

Additionally, we built a system with the crystal structure of KcsA T75A in the open state [PDB ID 6by3, (25)], and a control system with this structure back-mutated to WT KcsA. Briefly, we introduced all mutations outside the SF necessary to match the sequence of other systems we simulated, and built the two systems in CHARMM-GUI. Each of the final systems contained 39 DOPG, 105 DOPE, 7,700 water molecules, and 114 K^+^ (0.8 M) and 91 Cl^−^ for an electrically neutral system.

After building a system in CHARMM-GUI, it was equilibrated using the default six-step protocol and CHARMM36m/TIP3P parameters (27, 55–57) provided by CHARMM-GUI to reach the temperature of 290 K and the pressure of 1 bar. After introducing necessary mutations, the systems were additionally equilibrated for up to 100 ns in the NPT ensemble at respective temperatures and pressure [in this and all the subsequent simulations kept constant by the semi-isotropic barostat Parrinello–Rahman barostat (58) and velocity rescale (v-rescale) thermostat (59)], without restraints. Periodic boundary conditions (xyz) were used. Constraints were used for bonds with H atoms [LINCS (60)]. Simulation timestep was 2 fs. Lennard–Jones interactions were treated with a cutoff (forces switched to 0 from 1.0 to 1.2 nm in CHARMM36m, plain cutoff at 0.9 nm with long-range dispersion corrections for energy and pressure in Amber14sb). Coulomb interactions were treated with PME (61) with a 1.2-nm cutoff in CHARMM36m and 0.9 nm in Amber14sb. All simulations were carried out using GROMACS 2020.6 or GROMACS 2020.7 (62).

Amber14sb/TIP3P systems with LIPID17 lipids (28, 55, 63–65) were generated from the CHARMM36m structure immediately after the initial six-step equilibration, using the charmmlipid2amber (64, 65), GROMACS pdb2gmx, or CHARMM-GUI force field conversion tools (66). Systems in Amber14sb were then additionally equilibrated for up to 100 ns in the NPT ensemble before turning on the voltage, similar to the CHARMM36m systems.

Systems with Na^+^ as the permeating ion were prepared by either (i) replacing K^+^ with Na^+^, or (ii) with specific K^+^/Na^+^ ratios in select systems to simulate biionic conditions in the structures after equilibration.

### MD simulations with applied voltage

For production runs, a constant electric field was applied to the equilibrated structures along the *z*-axis (perpendicular to the membrane plane) to yield approximately 300 mV (or −300 mV for control simulations under negative voltage). Previously, it was shown that to obtain a correct transmembrane voltage in MD simulations, the whole box size in *z* should be taken into account (67). Correspondingly, the electric field *E* was calculated using a formula *E = ΔV/d_z_*, where *d_z_* is the size of the box along the *z-*axis. For each system, 10 simulation replicas were carried out with 1–2 μs per replica. For certain mutant systems, we performed control simulations starting from both direct and soft knock-on-like SF configurations. To check whether the channel stayed in the open state, we monitored the stability of the channel's activation gate by calculating the distances between T112 CA atoms of opposing subunits; the gate remained open for the majority of simulation time (Fig. S17). All simulations we carried out are listed in Table S1.

### Data analysis

To count permeation events, a custom Python script was used, kindly provided by Vytautas Gapsys (available in the shared data). The simulation box was divided into four regions—below the SF (“1”), in the SF but below its midpoint (“2”), in the SF above its midpoint (“3”), and above the SF (“4”). A permeation event was defined as a sequential transition 1→2→3→4 for outward permeation and in the opposite direction for inward permeation (i.e. ions that have been initially inside the SF are not counted, unless they reentered the SF later and then permeation occurred according to our definition). To calculate outward conductance, the difference between the number of outward and inward permeation events was used; accordingly, the inward conductance for systems at negative voltage was taken as the negative of that value. Presented conductance values are averages over all simulation replicas for a given system, with error bars representing 95% CIs calculated using Student's t-distribution.

To collect ion/water positions along the *z-*axis in SF, a custom FORTRAN code was used, available in (39). Dihedral angles were collected using the standard GROMACS toolbox. A flipped state of V76 was defined as the N–CA–C–O dihedral angle falling between −130° and −50°. For plotting the data, Python 3, matplotlib, and numpy were used (68, 69). Molecular visualizations were rendered using VMD (70).

## Supplementary Material

pgae272_Supplementary_Data

## Data Availability

Input files used to run MD simulations, example trajectories and the code used to count ion permeation events are publicly available at https://zenodo.org/doi/10.5281/zenodo.11083555.

## Preprints

This manuscript was posted on a preprint server: https://www.biorxiv.org/content/10.1101/2023.04.17.537168v1.
