# Supplementary material for: Selectivity filter mutations shift ion permeation mechanism in potassium channels
Source: PNAS Nexus. 2024 Jul 5;3(7):pgae272. doi: 10.1093/pnasnexus/pgae272 (PMC11251424; doi:10.1093/pnasnexus/pgae272)
Supplement: pgae272_Supplementary_Data [file pgae272_supplementary_data.pdf]

SI Figures

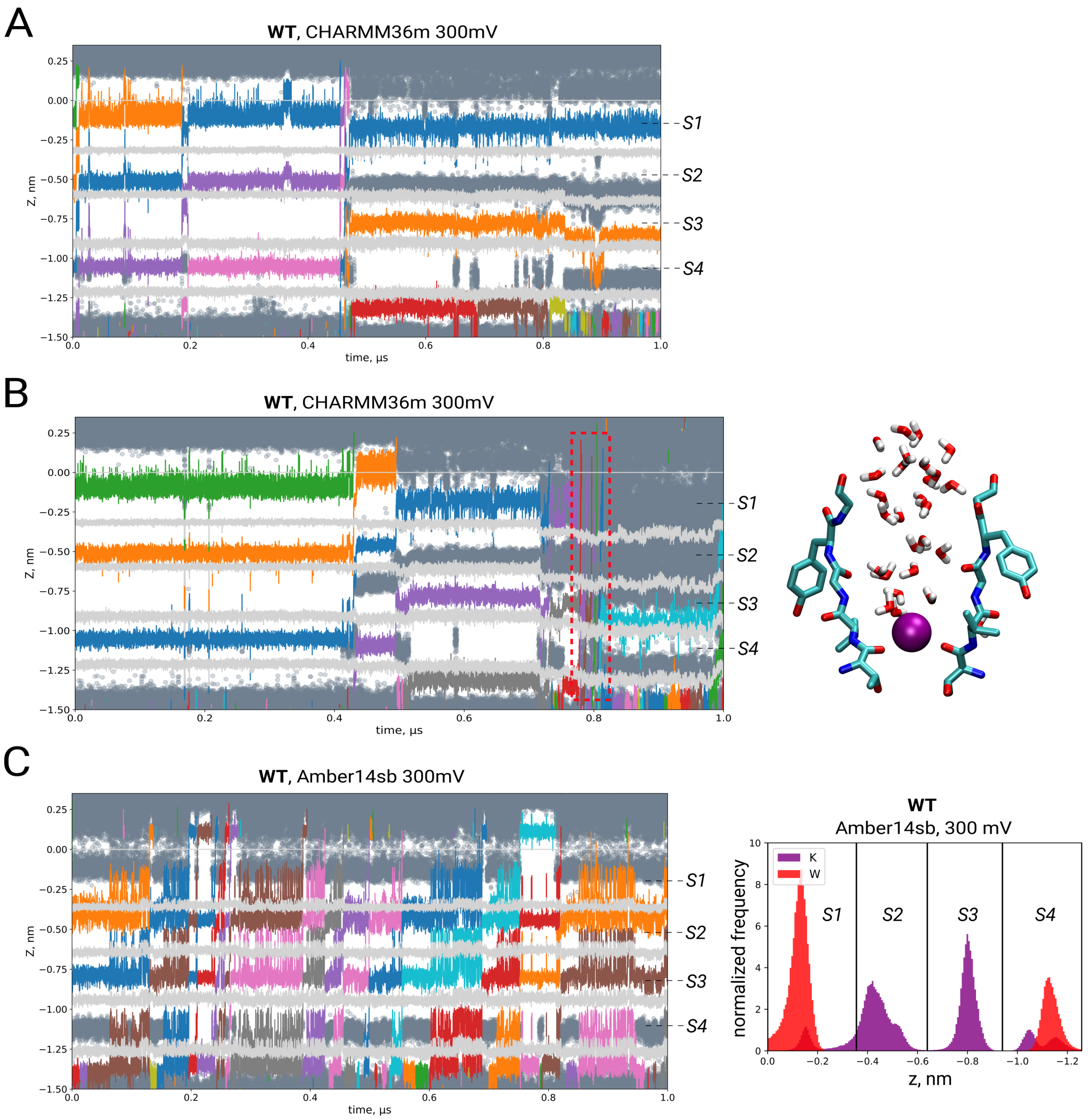

SI Fig. 1. (*A*) Example traces of K^+ ions (colored lines), water molecules (dark gray scatter) and backbone carbonyl oxygens/threonine hydroxyls (light gray lines) of the SF of WT KcsA in CHARMM36m showing K^+ permeation via direct knock-on followed by entrance of water into the SF and block of further K^+ permeation. (*B*) Traces showing several events of K^+/water co-permeation in one of the simulation replicas of WT KcsA in CHARMM36m (left). Those events were accompanied by a largely distorted SF with widening in the upper regions (right). (*C*) Traces showing K^+ permeation in WT in Amber14sb (left). Permeation occurred strictly via direct knock-on with water being virtually absent in the central regions of the SF, as demonstrated by the K^+/water distribution from all simulation replicas (right).

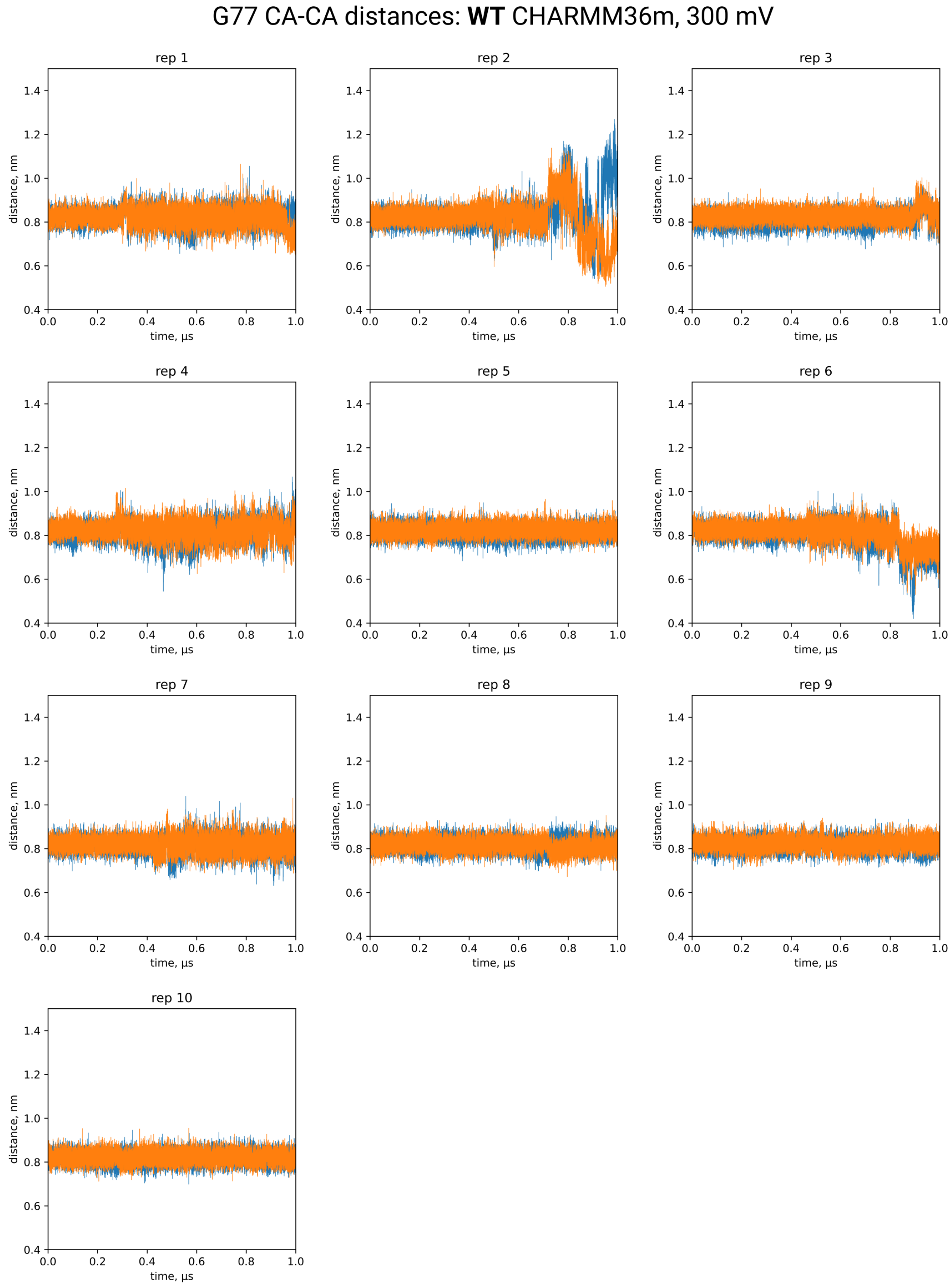

SI Fig. 2. Distance between α-carbons of G77 (opposing subunits) in the SF of WT KcsA. Transition to the inactivated state in WT KcsA is characterized by narrowing of the SF at G77 from ~0.8 nm to ~0.5 nm; in our simulations the WT SF was generally stable against this transition.

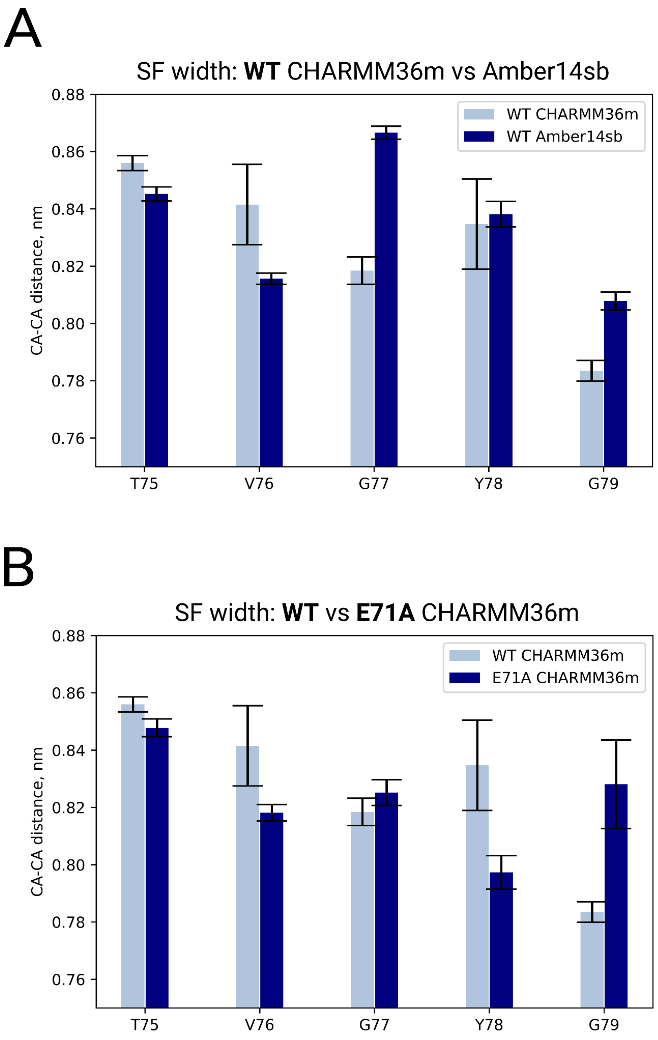

SI Fig. 3. Average distance between α-carbons of opposing subunits in the SF of WT KcsA in CHARMM36m and Amber14sb. Mean distances were calculated for each of 10 replicas, then the averages of these means were calculated; error bars represent CI 95% of those averages.

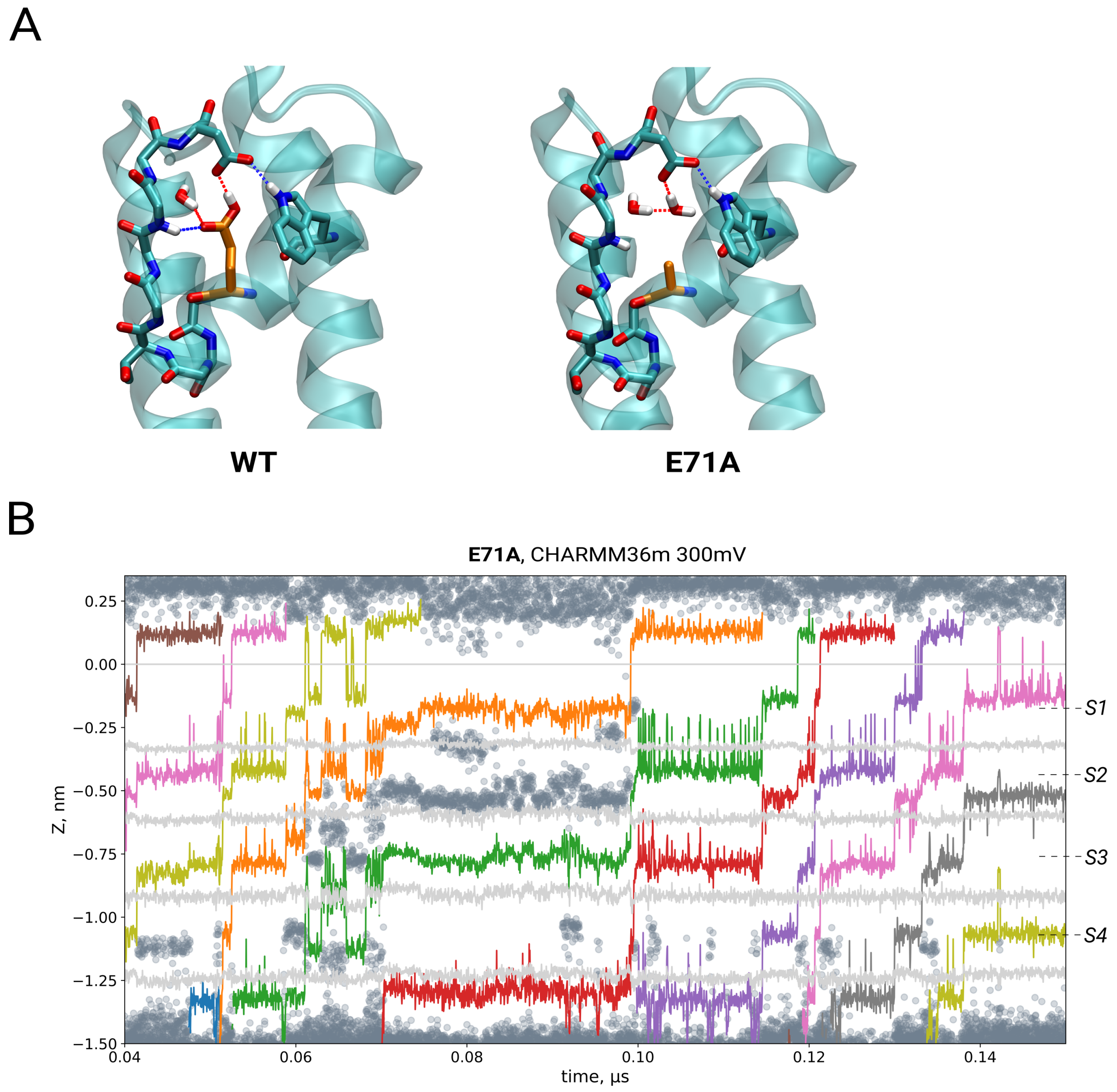

SI Fig. 4. (*A*) Hydrogen bond network behind the SF of WT KcsA (left, PDB ID 1k4c (1)) essential for SF inactivation. Mutation of E71 (orange) to alanine disrupts this hydrogen bond network (right, PDB ID 5vk6 (2)) and prevents the transition to the inactivated state. (*B*) Traces of K^+ ions (colored lines), water molecules (dark gray scatter) and backbone carbonyl oxygens/ threonine hydroxyls (light gray lines) of the SF in one of the simulation replicas of E71A in CHARMM36m show transient entrance of water into the SF, followed by continuation of ion permeation via water-free direct knock-on.

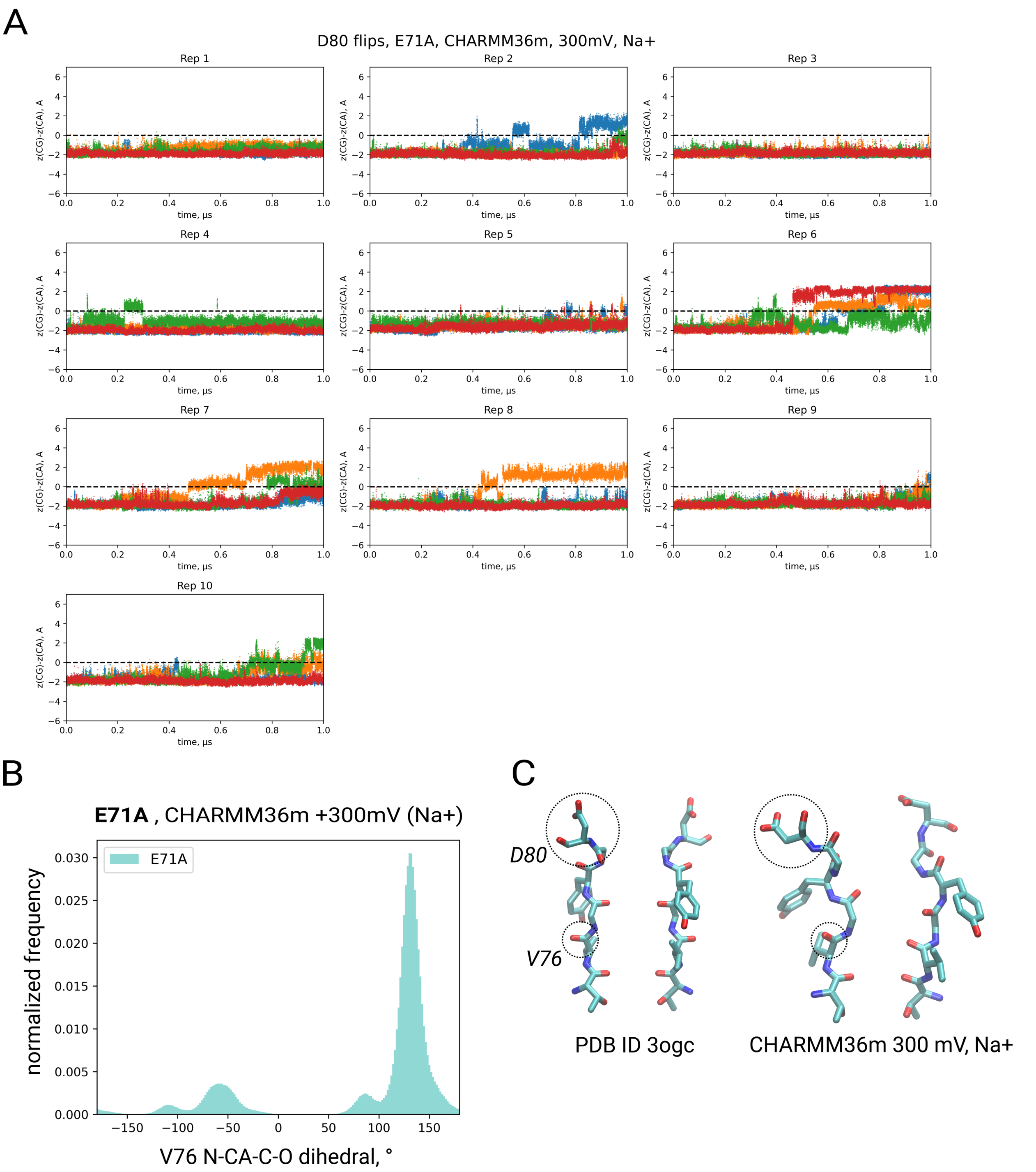

SI Fig. 5. (*A*) Traces of differences between z positions of CG and CA atoms of D80 for KcsA E71A, CHARMM36m, 300 mV, in presence of Na^+ but not K^+. Positions above the dashed line indicate a flipped D80. Colors correspond to different subunits. (*B*) Distribution of V76 N-CA-C-O dihedrals, the region from -130 to -50 corresponds to a flipped state. (*C*) The SF of KcsA E71A in the crystal structure obtained in Na^+-only conditions (left), and an example conformation with some D80 and V76 flipped as seen in our simulations (right).

A

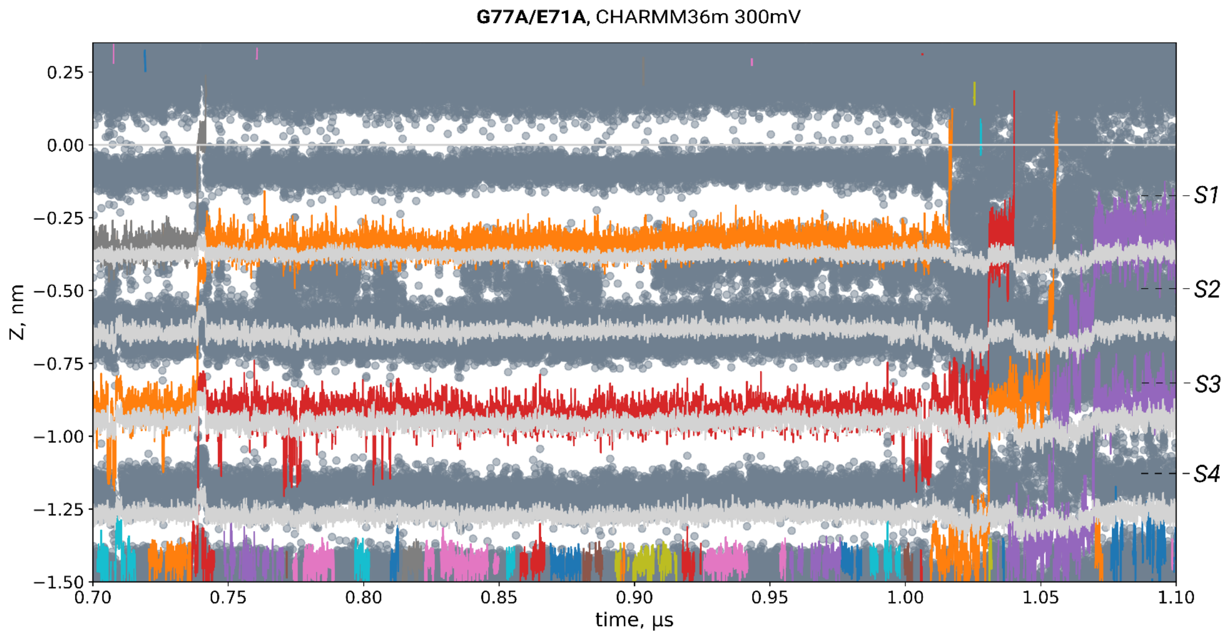

B

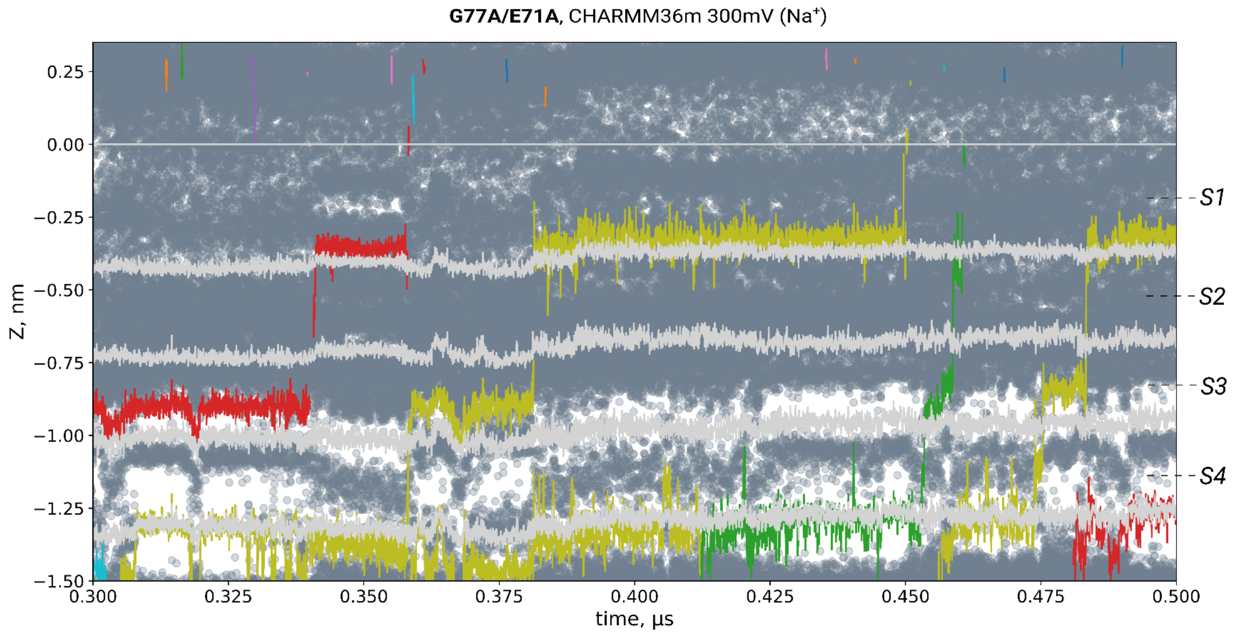

SI Fig. 6. (*A*) K^+ (colored lines), water (dark gray scatter) and backbone carbonyl oxygens'/ threonine hydroxyls traces (light gray lines) in the SF of G77A/E71A in CHARMM36m show permeation as jumps between KWKW-like configurations, without an intermediate WKWK. (*B*) Na^+ ion permeation in G77A/E71A in CHARMM36m.

A

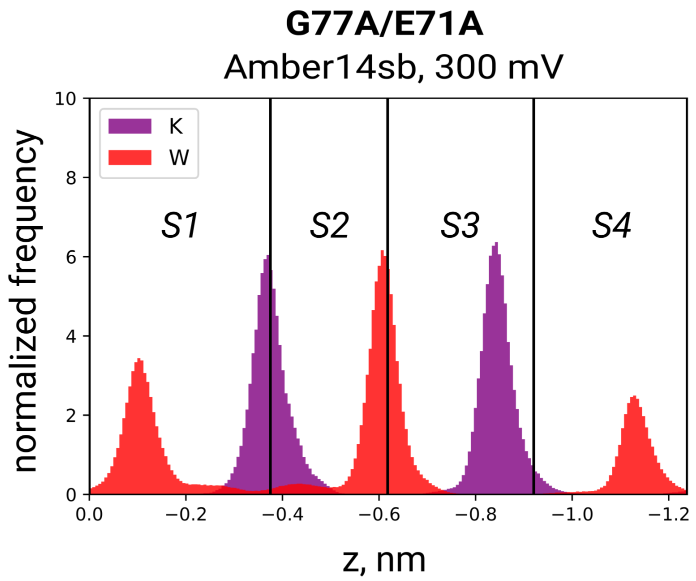

B

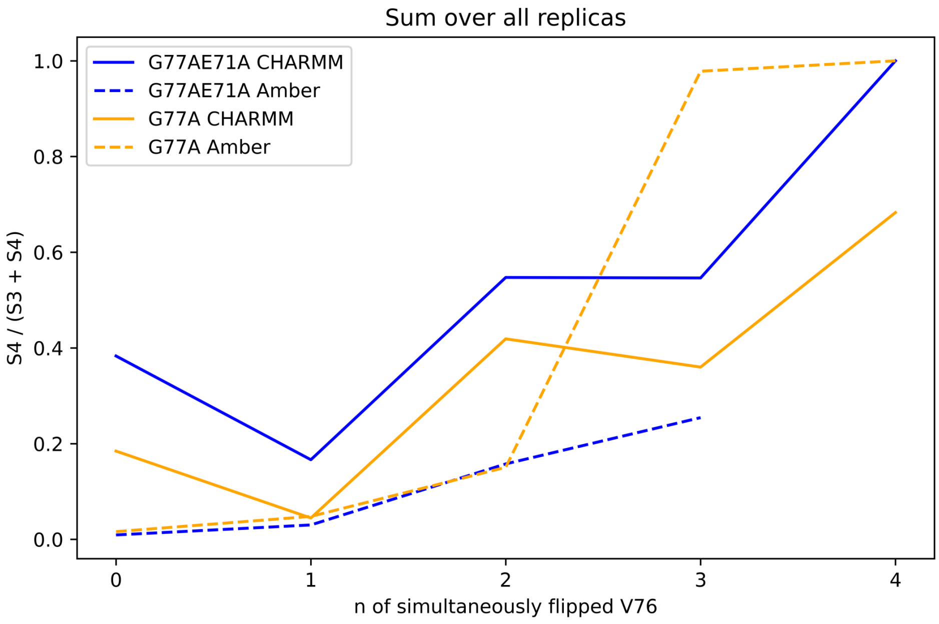

SI Fig. 7. (*A*) Distribution of K^+ and water in the SF of G77A/E71A, Amber14sb (all simulation replicas at 300 mV). (*B*) Fraction of K^+ in S4 opposed to S3 for n (0-4) simultaneously flipped V76 carbonyls for G77A/E71A and G77A, showing the tendency of K^+ to prefer the crystallographic S4 when more carbonyls are flipped.

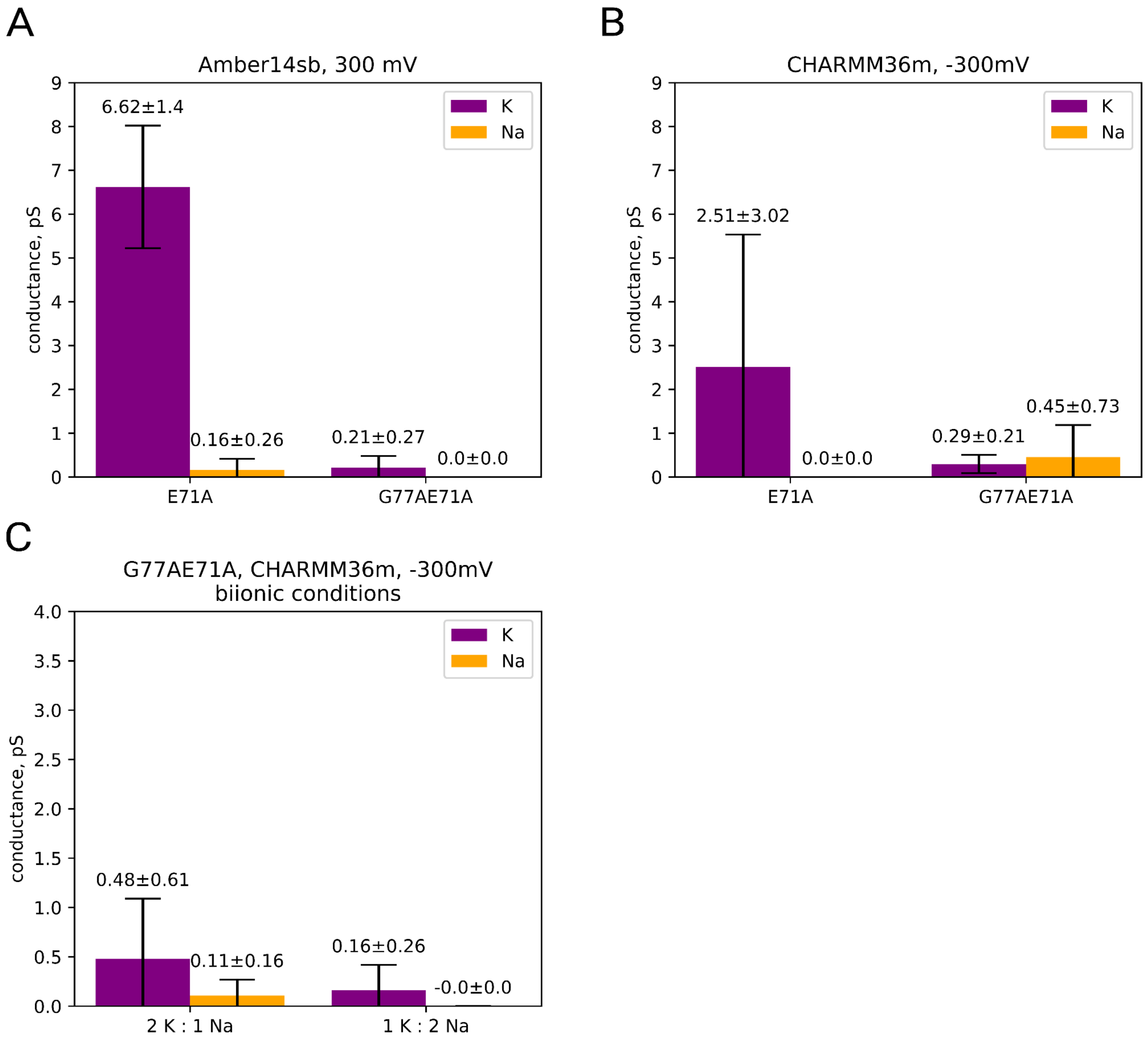

SI Fig. 8. K^+ and Na^+ conductances of (*A*) E71A and G77A/E71A in Amber14sb, (*B*) E71A and G77A/E71A in CHARMM36m at negative voltage, (*C*) G77A/E71A in CHARMM36m at negative voltage when both K^+ and Na^+ are present at respective concentration ratios.

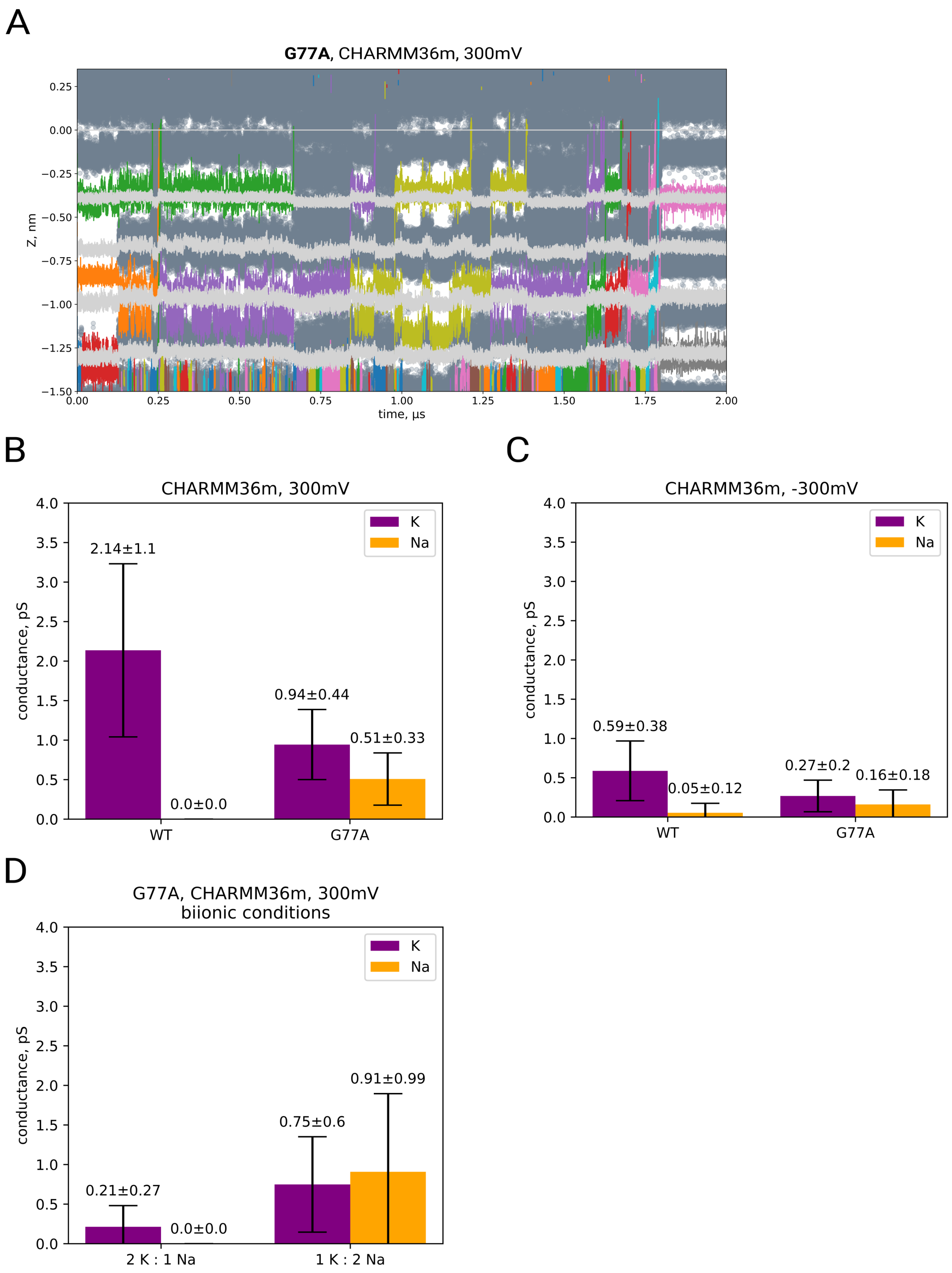

SI Fig. 9. (*A*) Co-permeation of K^+ (colored lines) and water (dark gray scatter) in the SF of G77A in CHARMM36m. Positions of backbone carbonyl oxygens and threonine hydroxyls are shown as light gray lines. K^+ and Na^+ conductances of WT and G77A in CHARMM36m at (*B*) positive and (*C*) negative voltages. (*D*) K^+ and Na^+ conductances of G77A in CHARMM36m at positive voltage and with both ions present at given concentration ratios.

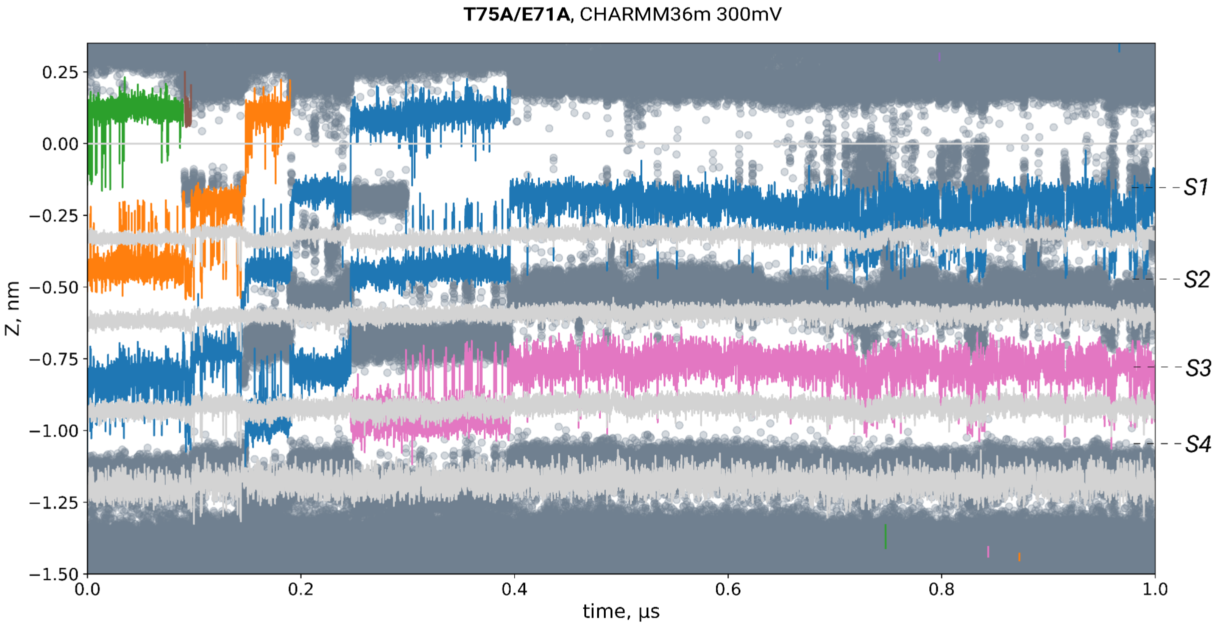

SI Fig. 10. Co-permeation of K^+ (colored lines) and water (dark gray scatter) in the SF of T75A/E71A in CHARMM36m. Positions of backbone carbonyl oxygens and one of the hydrogens on the CB atom of A75 (instead of the T75 hydroxyls) are shown as light gray lines.

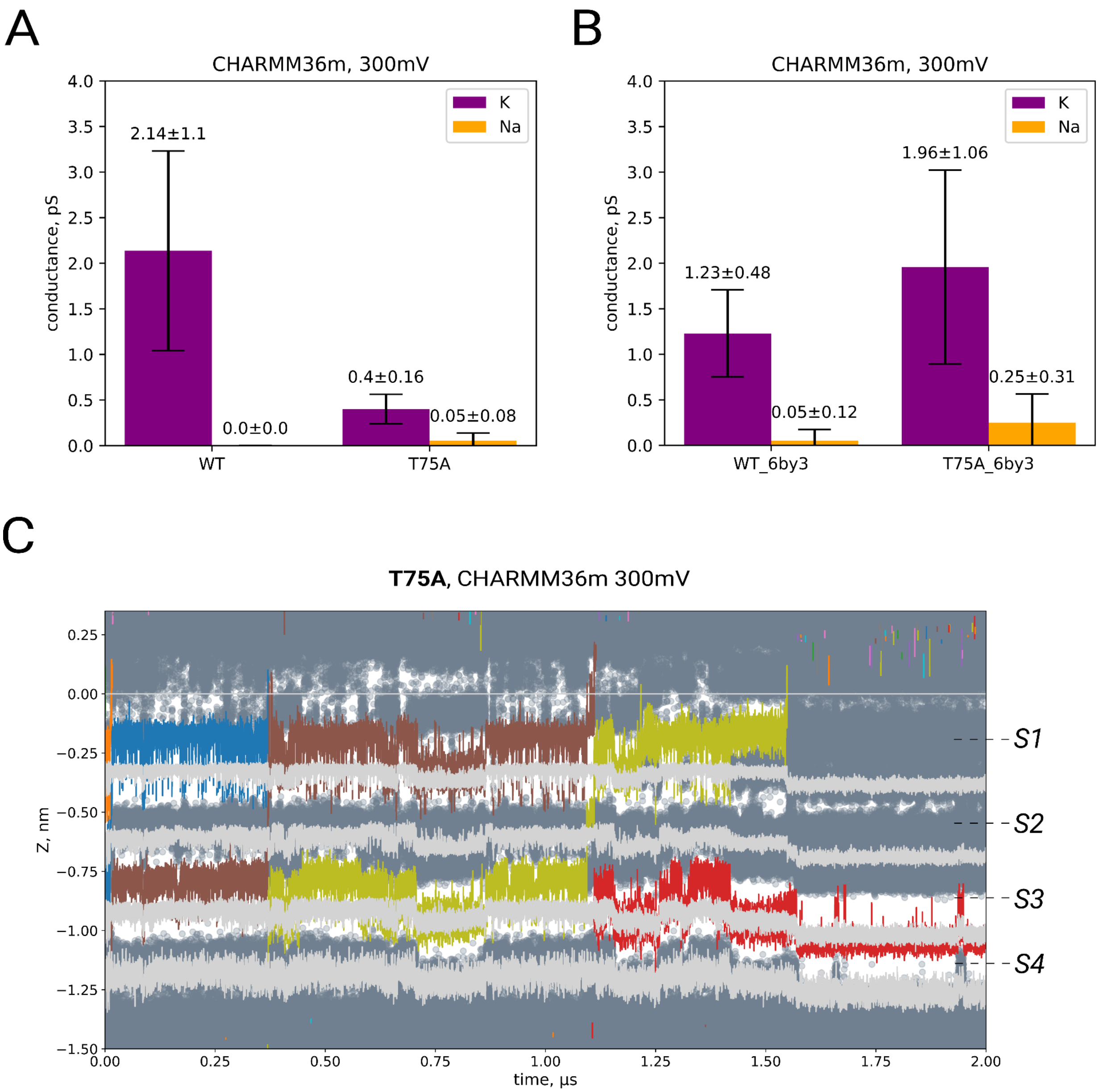

SI Fig. 11. (*A*) K^+ and Na^+ conductances of WT and T75A at 300 mV in CHARMM36m from the main simulation set, and (*B*) derived from the crystal structure of the open state of KcsA T75A (PDB ID 6by3 (3)). (*C*) Traces of K^+ (colored lines), water (dark gray scatter) and backbone carbonyl oxygens/one of the hydrogens on the CB atom of A75 instead of the T75 hydroxyls (light gray lines) in the SF of T75A from the main simulation set in CHARMM36m.

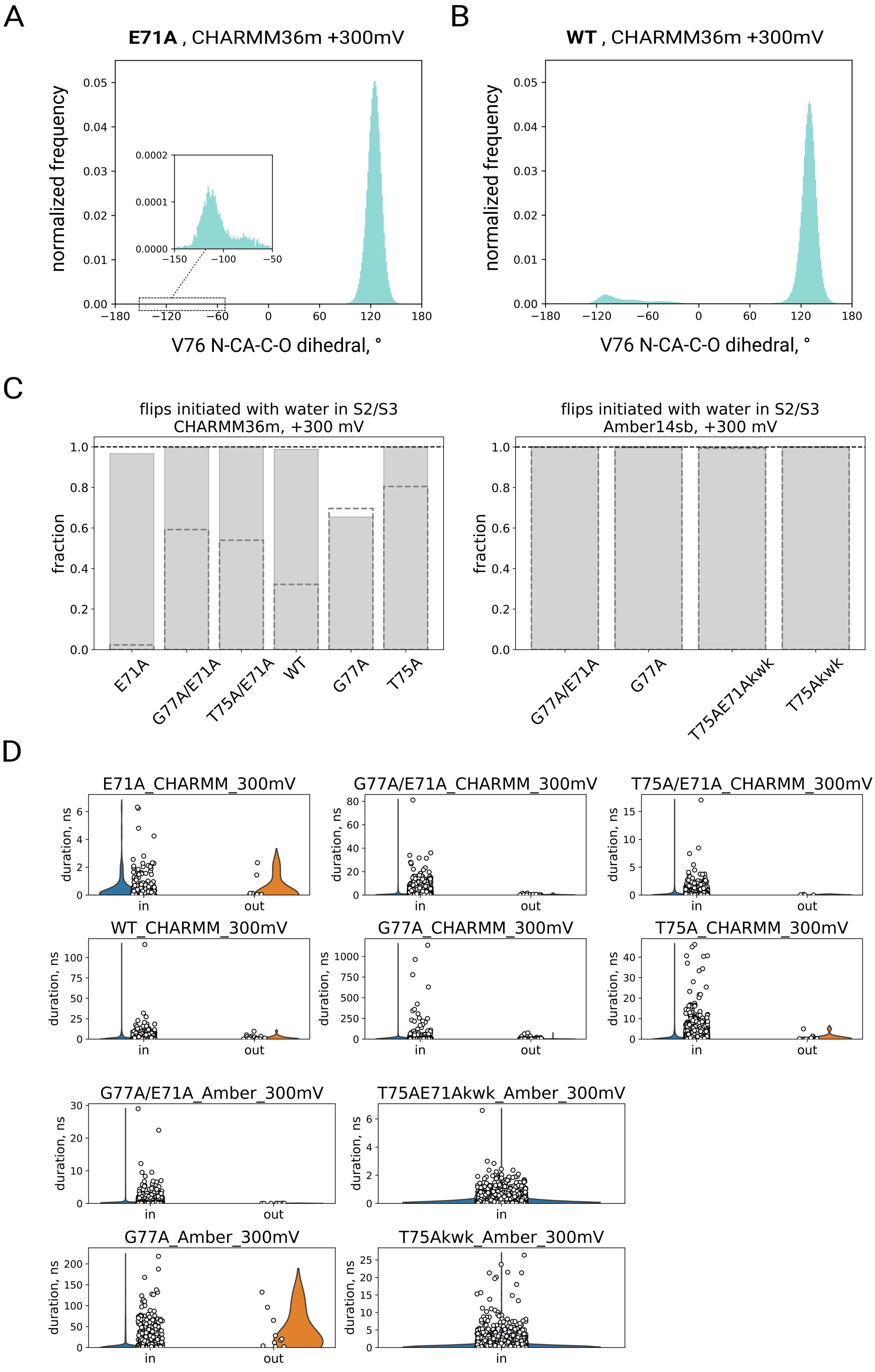
A
E71A , CHARMM36m +300mV
normalized frequency
V76 N-CA-C-O dihedral, °
B
WT , CHARMM36m +300mV
normalized frequency
V76 N-CA-C-O dihedral, °
C
flips initiated with water in S2/S3
CHARMM36m, +300 mV
fraction
E71A
G77A/E71A
T75A/E71A
WT
G77A
T75A
flips initiated with water in S2/S3
Amber14sb, +300 mV
fraction
G77A/E71A
G77A
T75AE71Akwk
T75Akwk
D
E71A_CHARMM_300mV
G77A/E71A_CHARMM_300mV
T75A/E71A_CHARMM_300mV
WT_CHARMM_300mV
G77A_CHARMM_300mV
T75A_CHARMM_300mV
G77A/E71A_Amber_300mV
T75AE71Akwk_Amber_300mV
G77A_Amber_300mV
T75Akwk_Amber_300mV
duration, ns
in
out

SI Fig. 12. Both E71A (*A*) and WT KcsA (*B*) show low frequency of V76 carbonyl flipping, as shown by distributions of V76 N-CA-C-O dihedrals. (*C*) Solid bars represent fractions of flipping events initiated when water was already present in SF sites S2/S3 out of all flipping events. A flipping event is defined as a continuous stretch of simulation where at least one V76 is flipped. Systems where flips weren't detected are not shown. Dashed bars represent the fraction of frames with water in S2/S3 out of all simulation frames; as water is not always present in S2/S3 in CHARMM36m (*left*), but the majority of flips still happen from the water-filled SF, this suggests that water causes V76 flips. In Amber14sb, establishing a causal relationship was more difficult, as the SF always contained water molecules. (*D*) Distribution of durations of flipping events that started when water was already in S2/S3 ('in') and when no water was present in these sites ('out').

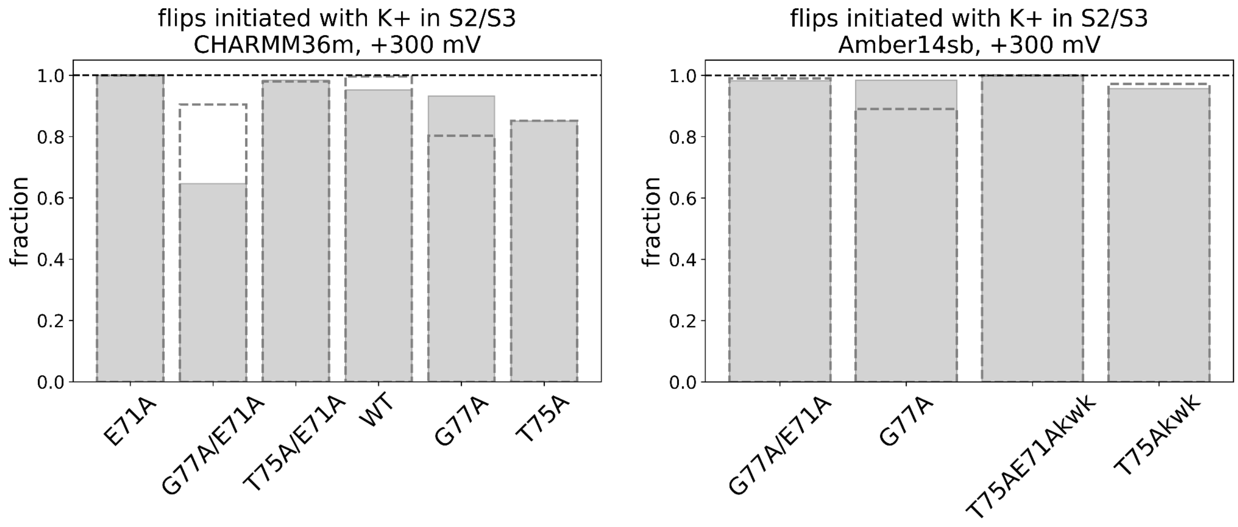

SI Fig. 13. Fractions of flipping events initiated when K^+ was present in SF sites S2/S3 out of all flipping events. Systems where flips haven't been detected are not shown. In Amber14sb flips occurred only when soft knock-on-like starting SF configurations were used ('kwk'). Bars with dashed edges represent the fraction of frames where K^+ were in S2/S3, out of all simulation frames.

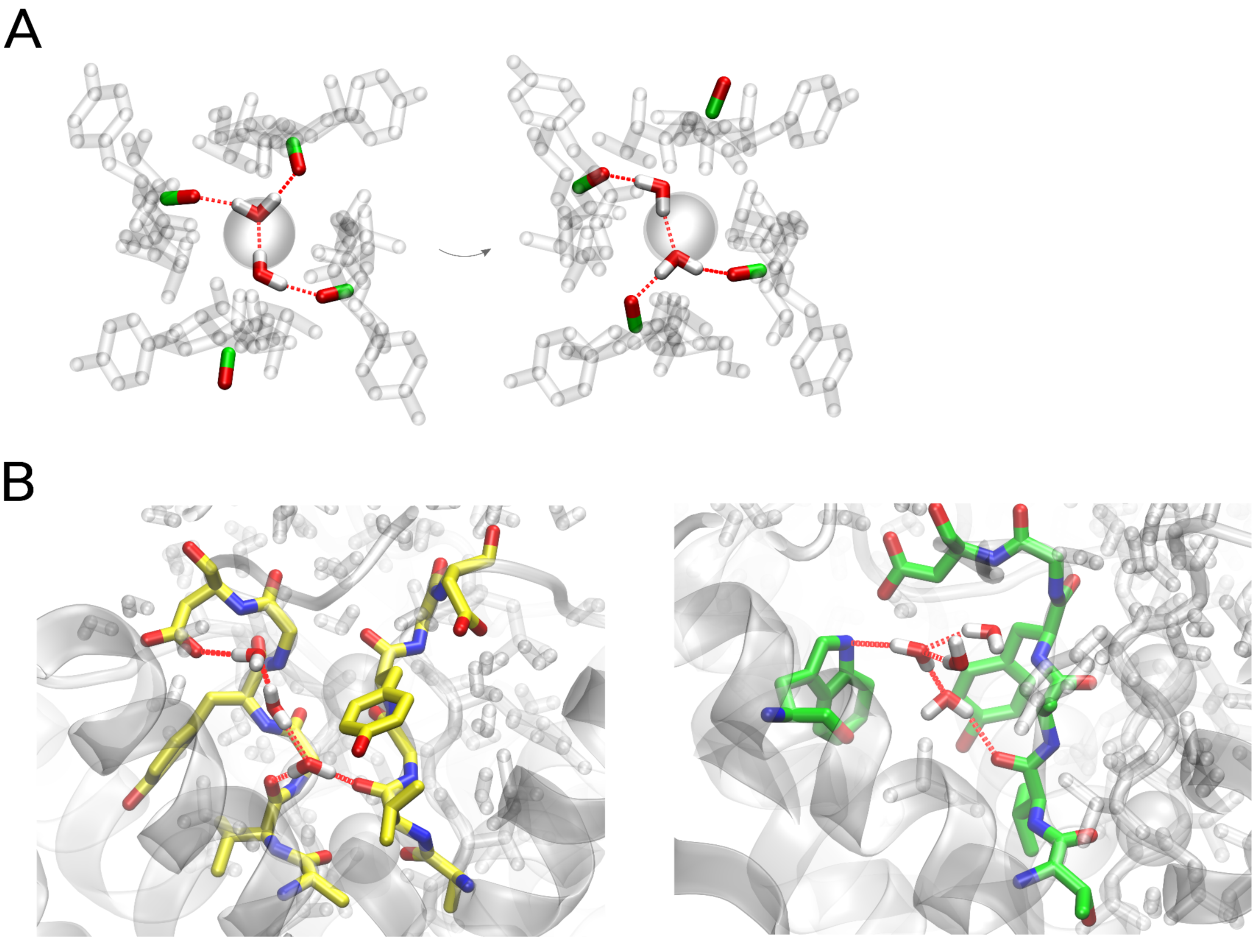

SI Fig. 14. (*A*) Possible mechanism of stabilization of flipped and non-flipped V76 carbonyls through specific hydrogen bond networks when water molecules are present in S2 or S3. Water molecules and V76 carbonyls are opaque. Snapshots show a rearrangement of the network accompanied by a flipping of one carbonyl, and unflipping of another. (*B*) Stabilization of flips by hydrogen bond networks involving water molecules behind the SF in T75A/E71A (left) and G77A/E71A (right).

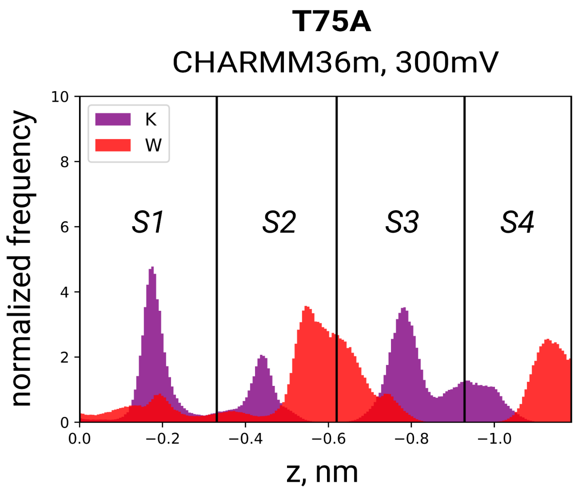

SI Fig. 15. Distribution of K^+ and water in the SF of T75A in CHARMM36m (all simulation replicas at 300 mV).

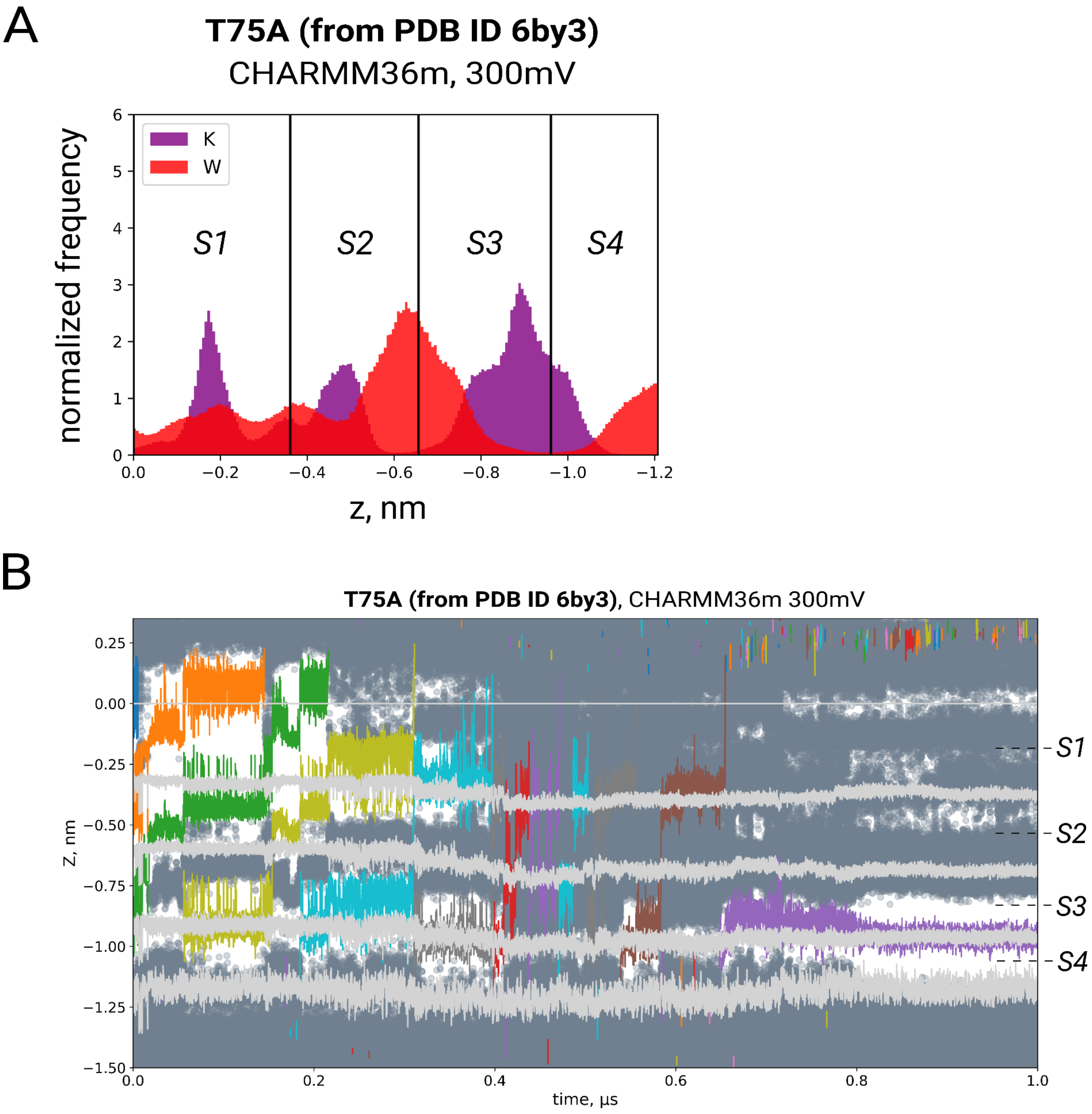

SI Fig. 16. (*A*) K^+/water distribution and (*B*) traces showing K^+ (colored lines) and water (gray scatter) permeation in the SF (backbone carbonyls and one of the hydrogens on the CB atom of A75 (instead of the T75 hydroxyls) are represented by light gray lines) of T75A built from the crystal structure of the open state of KcsA T75A (PDB ID 6by3 (3)).

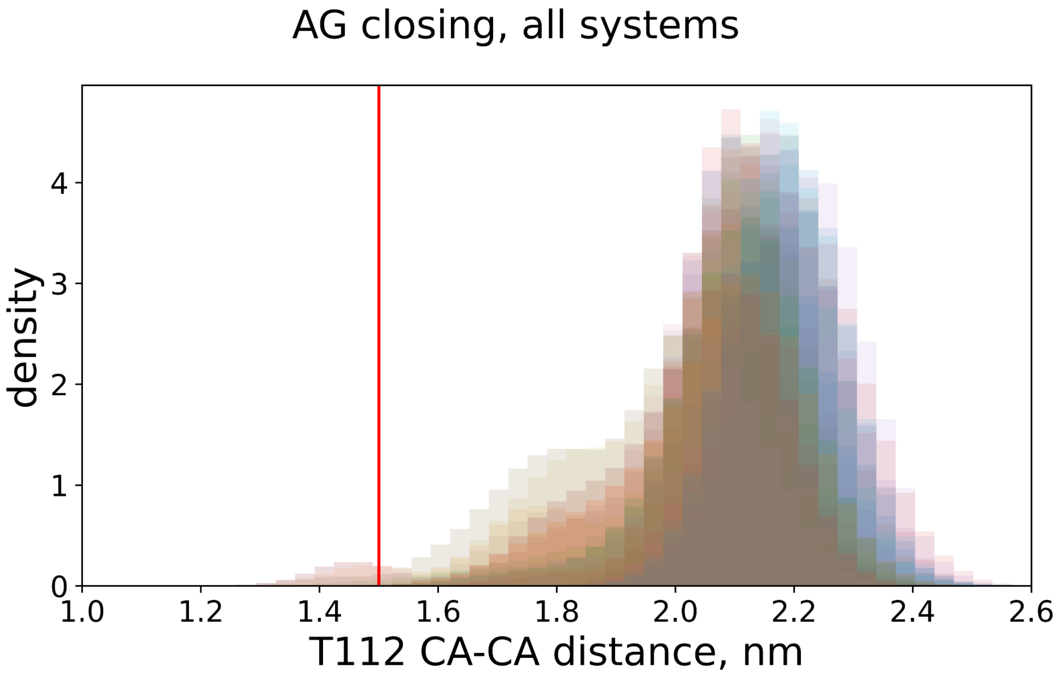

SI Fig. 17. Distribution of distances between T112 CA atoms of opposite subunits in all systems and conditions as a measure of the state of the activation gate. Red line indicates the activation gate opening in the partially open state (~1.5 nm, PDB ID 3fb5 (4)) below which we considered the gate to be closed. The channel remained in the open state for the majority of simulation time.

SI Table 1. List of all simulated systems. 'KK' and 'KWK' (or 'NaNa' and 'NaWNa') indicate whether a direct or a soft knock-on-like initial configuration was used (containing K^+ or Na^+ in the SF, respectively). For simulations in biionic conditions, ratios of Na^+ and K^+ concentrations are given. Unless stated, all systems are built from PDB ID 5vk6.

System	Force field	Voltage	Permeant ions	Initial SF configuration	Simulation time	Permeation events
E71A	CHARMM36m	300 mV	K^+	KK	10x1 µs	494
			Na^+	NaNa	10x1 µs	0
		-300 mV	K^+	KK	10x1 µs	47
			Na^+	NaNa	10x1 µs	0
	Amber14sb	300 mV	K^+	KK	10x1 µs	124
			Na^+	NaNa	10x1 µs	3
WT	CHARMM36m	300 mV	K^+	KK	10x1 µs	40
			Na^+	NaNa	10x1 µs	0
		-300 mV	K^+	KK	10x1 µs	11
			Na^+	NaNa	10x1 µs	1
	Amber14sb	300 mV	K^+	KK	10x1 µs	81
			Na^+	NaNa	10x1 µs	2
WT (from PDB ID 6by3)	CHARMM36m	300 mV	K^+	KK	10x1 µs	23
			Na^+	NaNa	10x1 µs	1
G77A/E71A	CHARMM36m	300 mV	K^+	KK	10x~2 µs (total 19.8 µs)	15
			K^+	KWK	10x1 µs	1
			Na^+	NaNa	10x2 µs	42
			1 Na^+ : 2 K^+	KK	10x1 µs	K: 0, Na: 0
			2 Na^+ : 1 K^+	KK	10x1 µs	K: 0, Na: 0
		-300 mV	K^+	KK	10x2 µs	11

			Na^+	NaNa	10x2 µs	17
			1 Na^+ : 2 K^+	KK	10x1 µs	K: 9, Na: 2
			2 Na^+ : 1 K^+	KK	10x1 µs	K: 3, Na: 0
	Amber14sb	300 mV	K^+	KWK	10x2 µs	4
			Na^+	NaWNa	10x2 µs	0
			1 Na^+ : 2 K^+ *	KWK	10x1 µs	K: 1, Na: 0
			2 Na^+ : 1 K^+ *	KWK	10x1 µs	K: 0, Na: 0
G77A	CHARMM36m	300 mV	K^+	KK	10x2 µs	33
			K^+	KWK	5x1 µs	10
			Na^+	NaNa	10x2 µs	19
			1 Na^+ : 2 K^+	KK	10x1 µs	K: 4, Na: 0
			2 Na^+ : 1 K^+	KK	10x1 µs	K: 14, Na: 17
		-300 mV	K^+	KK	10x1 µs	5
			Na^+	NaNa	10x1 µs	3
	Amber14sb	300 mV	K^+	KWK	10x1 µs	0
			Na^+	NaWNa	10x1 µs	0
T75A/E71A	CHARMM36m	300 mV	K^+	KK	10x1 µs	10
			Na^+	KK	10x1 µs	8
			Na^+	NaWNa	5x1 µs	2
		-300 mV	K^+	KK	10x1 µs	0
			Na^+	NaNa	10x1 µs	0
	Amber14sb	300 mV	K^+	KK	10x1 µs	0
			K^+	KWK	5x1 µs	0
			Na^+	NaNa	10x1 µs	0
			Na^+	NaWNa	5x1 µs	0

T75A	CHARMM36m	300 mV	K^+	KK	10x2 μs	15
			Na^+	NaNa	10x2 μs	1
			Na^+	NaWNa	5x1 μs	1
		-300 mV	K^+	KK	10x1 μs	0
			Na^+	NaNa	10x1 μs	0
	Amber14sb	300 mV	K^+	KK	10x1 μs	0
			K^+	KWK	5x1 μs	0
			Na^+	NaNa	10x1 μs	0
T75A (PDB ID 6by3)	CHARMM36m	300 mV	K^+	KK	20x1 μs	61
			Na^+	NaNa	20x1 μs	8

** - performed with the CHARMM36m cut-off scheme; however, as the system in these conditions had practically no ion permeation, the conclusions are not affected*

References

1. Y. Zhou, J. H. Morais-Cabral, A. Kaufman, R. MacKinnon, Chemistry of ion coordination and hydration revealed by a K+ channel-Fab complex at 2.0 A resolution. *Nature* **414**, 43–48 (2001).

2. L. G. Cuello, D. M. Cortes, E. Perozo, The gating cycle of a K+ channel at atomic resolution. *Elife* **6** (2017).

3. A. J. Labro, D. M. Cortes, C. Tilegenova, L. G. Cuello, Inverted allosteric coupling between activation and inactivation gates in K+ channels. *Proc. Natl. Acad. Sci. U. S. A.* **115**, 5426–5431 (2018).

4. L. G. Cuello, V. Jogini, D. M. Cortes, E. Perozo, Structural mechanism of C-type inactivation in K(+) channels. *Nature* **466**, 203–208 (2010).
